# Diagnostic Accuracy of Transvaginal Ultrasound and Magnetic Resonance Imaging for the Detection of Myometrial Infiltration in Endometrial Cancer: A Systematic Review and Meta-Analysis

**DOI:** 10.3390/cancers16050907

**Published:** 2024-02-23

**Authors:** István Madár, Anett Szabó, Gábor Vleskó, Péter Hegyi, Nándor Ács, Péter Fehérvári, Tamás Kói, Emma Kálovics, Gábor Szabó

**Affiliations:** 1 Centre for Translational Medicine, Semmelweis University, 1088 Budapest, Hungary; madar.istvan@semmelweis.hu (I.M.); vleskogabor@gmail.com (G.V.); hegyi.peter@semmelweis.hu (P.H.); acs.nandor@semmelweis.hu (N.Á.); peter.fehervari.tmk@gmail.com (P.F.); samatiok@gmail.com (T.K.); kalovicsemma@gmail.com (E.K.); 2 Department of Obstetrics and Gynecology, Semmelweis University, 1088 Budapest, Hungary; 3 Department of Urology, Semmelweis University, 1082 Budapest, Hungary; 4 Institute for Translational Medicine, Medical School, University of Pécs, 7624 Pécs, Hungary; 5 Institute of Pancreatic Diseases, Semmelweis University, 1083 Budapest, Hungary; 6 Department of Biostatistics, University of Veterinary Medicine, 1078 Budapest, Hungary; 7 Stochastics Department, Budapest University of Technology and Economics, 1111 Budapest, Hungary

**Keywords:** endometrial neoplasm, endometrium carcinoma, ultrasonography, ultrasonographic imaging, MRI scan, preoperative staging, low-grade endometrial cancer, diagnostic imaging, comparative analysis, head-to-head analysis

## Abstract

**Simple Summary:**

The optimal imaging method for deep myometrial infiltration assessment in endometrial cancer is uncertain. We aimed to compare transvaginal ultrasound and magnetic resonance imaging in the preoperative assessment of deep myometrial infiltration. Our study indicates that transvaginal ultrasound provides diagnostic performance comparable to magnetic resonance imaging. However, magnetic resonance imaging showed significantly better specificity in low-grade endometrial cancer. Further studies are needed for the evaluation of myometrial infiltration, especially concerning patients with fertility-sparing wishes.

**Abstract:**

In endometrial cancer (EC), deep myometrial invasion (DMI) is a prognostic factor that can be evaluated by various imaging methods; however, the best method of choice is uncertain. We aimed to compare the diagnostic performance of two-dimensional transvaginal ultrasound (TVS) and magnetic resonance imaging (MRI) in the preoperative detection of DMI in patients with EC. Pubmed, Embase and Cochrane Library were systematically searched in May 2023. We included original articles that compared TVS to MRI on the same cohort of patients, with final histopathological confirmation of DMI as reference standard. Several subgroup analyses were performed. Eighteen studies comprising 1548 patients were included. Pooled sensitivity and specificity were 76.6% (95% confidence interval (CI), 70.9–81.4%) and 87.4% (95% CI, 80.6–92%) for TVS. The corresponding values for MRI were 81.1% (95% CI, 74.9–85.9%) and 83.8% (95% CI, 79.2–87.5%). No significant difference was observed (sensitivity: *p* = 0.116, specificity: *p* = 0.707). A non-significant difference between TVS and MRI was observed when no-myometrium infiltration vs. myometrium infiltration was considered. However, when only low-grade EC patients were evaluated, the specificity of MRI was significantly better (*p* = 0.044). Both TVS and MRI demonstrated comparable sensitivity and specificity. Further studies are needed to assess the presence of myometrium infiltration in patients with fertility-sparing wishes.

## 1. Introduction

Endometrial cancer (EC) is the sixth most common cancer in women worldwide. In 2020 alone, 417,000 new cases and 97,000 deaths were reported worldwide attributed to EC [1]. Due to the increasing rate of obesity, the average age of populations and other risk factors, the overall incidence of EC has risen by 132% over the last 30 years [2].

In EC, hysterectomy and bilateral salpingo-oophorectomy are usually unavoidable. The extent of concomitant lymphadenectomy is determined by risk factors such as tumor grade, histological type, stage, myometrial infiltration depth, lymphovascular space invasion (LVSI) and molecular type. However, the choice of extended surgery is associated with an elevated risk of complications [3].

The ESGO/ESTRO/ESP (European Society of Gynaecological Oncology, European Society for Radiotherapy and Oncology, European Society of Pathology) guidelines for the management of patients with EC were published in 2021. The preoperative evaluation of EC should determine the histopathologic type, grade, myometrial invasion, and LVSI, preferably with the addition of molecular classification. On the basis of these criteria, five prognostic risk groups were determined (low, intermediate, high-intermediate, high, and advanced), where the presence of deep myometrial invasion (DMI) plays an important role. In patients with low- or intermediate-risk disease, sentinel lymph node biopsy can be a viable option for staging. However, in patients with high-intermediate or high-risk disease, surgical lymph node staging should be performed [4].

For DMI assessment, a transvaginal or transrectal ultrasound performed by an expert or a pelvic magnetic resonance imaging (MRI) is recommended [4,5,6,7]. The most recent meta-analysis comparing 2D-TVS (transvaginal ultrasound) to MRI was published in 2017 and concluded that MRI showed similar specificity and superior sensitivity compared to TVS in detecting DMI in women with EC, but the difference was not statistically significant [8]. Some authors suggest the usage of MRI only for patients in whom TVS, carried out by an expert, produces images of poor quality [9]. Currently, there is no consensus on the imaging method of choice between the different guidelines [7]. There are conflicting data in the literature regarding the utility of the intraoperative frozen section for DMI [10,11]. Hence, this is currently not recommended by the most recent ESGO/ESTRO/ESP guideline [4]. Three-dimensional transvaginal ultrasound does not seem to be a superior method compared to two-dimensional TVS in terms of diagnostic accuracy and interrater agreement [12,13,14].

Among individuals diagnosed with low-grade (grade 1 or 2 endometrioid) EC, approximately 15.4% are below the age of 50 years, while approximately 4.2% of women are diagnosed before the age of 40 years [15]. In 2023, the ESGO/ESHRE/ESGE (European Society of Gynaecological Oncology, European Society of Human Reproduction and Embryology, European Society for Gynaecological Endoscopy) guidelines for the fertility-sparing treatment of patients with EC were published. Accordingly, in patients with endometrioid EC with grade 1, stage IA tumors, without myometrial invasion and without other risk factors, the option of fertility-sparing treatment can be considered [16]. However, the assessment of transvaginal ultrasound or MRI in determining the absence of myometrial invasion or shallow myometrial invasion is based on extrapolation from data on diagnosing DMI [16].

Therefore, we aimed to investigate the diagnostic accuracy of TVS and MRI in the detection of DMI in EC.

## 2. Materials and Methods

In accordance with the PRISMA 2020 guidelines [17] (Table S1), we presented our systematic review and meta-analysis following the methodological principles outlined in the Cochrane Handbook [18]. Our study protocol was registered on PROSPERO, under registration number CRD42023426934.

### 2.1. Eligibility Criteria

We included prospective and retrospective cohort studies. The included population was EC patients who underwent hysterectomy and were examined preoperatively using both 2-dimensional (2D) TVS (Index Test 1) and MRI (Index Test 2), compared to definitive postoperative histology (Reference Test) as the gold standard for diagnosing myometrial invasion depth.

Studies (1) with inaccurate or inconsistent data or where data were presented in a way that could not be further processed, (2) conference abstracts, reviews, case series and case reports, (3) previous systematic reviews and meta-analyses, (4) articles containing data on patients screened exclusively with either TVS or MRI and (5) articles in which 3D ultrasound was the index test were excluded. Attempts were made to contact the authors of articles with inconsistent data.

Studies that reported on the level of myometrial invasion (<50% or ≥50%) were eligible for data processing. True positive, true negative, false positive and false negative values were all calculable or included in the articles.

No language or other restrictions were applied.

### 2.2. Information Sources and Search Strategies

Our systematic search was conducted in three databases: MEDLINE (Pubmed), Embase and Cochrane Library on 22 May 2023. The domains of the search key included endometrium, cancer, transvaginal sonography and magnetic resonance imaging. See the full search key in Section S1.

### 2.3. Selection and Data Collection Process

After duplicate removal, title-abstract and full-text selection processes were conducted by two independent authors (IM, GV). To assess inter-rater agreement, Cohen’s kappa coefficients (κ) were calculated at each step [19]. Disagreements were resolved by a third author (NÁ). Duplicate records were eliminated using EndNote 20 (Clarivate Analytics, Philadelphia, PA, USA), and the remaining articles were assessed using Rayyan [20]. Data were extracted into a predefined sheet. Notably, if both expert and non-expert data were provided for the same patient cohort, expert data were included in the final analysis.

### 2.4. Data Items

The following data were extracted: first author, year of publication, study population, study period, study type, settings (single/multicenter), patient data (total number, menopausal status, age), distribution of the patients with <50% or ≥50% of myometrial involvement, whether data on patients with low-grade EC were available, data on TVS and MRI: number of true positive, true negative, false positive, false negative patients, data on sensitivity and specificity.

### 2.5. Study Risk of Bias Assessment

The risk of bias assessment was performed independently by two authors (IM, GV), with disagreements resolved by a third author (GS). To evaluate the quality of the included studies, the modified version of the Quality Assessment of Diagnostic Accuracy Studies-2 (QUADAS-2) tool was used.

### 2.6. Synthesis Methods

Statistical analyses were performed using R statistical software (version 4.1.2) and the R script of the online tool described by Freeman [21]. For all statistical analyses, a *p*-value of less than 0.05 was considered significant.

Two by two contingency tables were directly extracted or calculated from the studies containing true positive, false positive, false negative and true negative values. To pool sensitivity and specificity, the bivariate models of Reitsma and Chu were fitted [22,23]. We used ROC (receiver-operating characteristic curve) plots to illustrate the sensitivities and specificities of the included studies, the summary estimates of sensitivity and specificity and the corresponding 95% confidence and prediction regions. The confidence region contained both pooled sensitivity and specificity (more specifically, 1-specificity) in 95% of the cases. The prediction region contained the true sensitivity and specificity (more exactly, 1-specificity) of a new study in 95% of the cases, thus providing excellent insight into heterogeneity. In these visualizations, the sizes of the ellipsoids reflected the weights of the studies, calculated according to the method described by Burke [24]. Besides the prediction region, heterogeneity was assessed by performing separate univariate analyses of sensitivity. Specifically, we used the generalized mixed-effect approach of Stijnen et al. and calculated the I^2^ measure and its confidence interval [25].

The statistical challenge was that the performance of the MRI and TVS was evaluated in the same population within each study, so we separately compared sensitivity and specificity as follows: Separately for the logit transformed sensitivity and specificity, we constructed a two-dimensional (with coordinates MRI logit sensitivity and TVS logit sensitivity, and for specificity analysis MRI logit specificity and TVS logit specificity) model using the rma.mv() function of the metafor R package. To circumvent the problem caused by the unknown correlations, we supplemented the method with the robust approach of Pustejovsky [26], implemented in the coef_test() function of the clubSandwhich R package. Moreover, we repeated the approach under several within-study correlation assumptions. All sensitivity runs provided similar *p*-values. We assessed possible time trends in the sensitivity and specificity values by performing a random effect meta-regression using year as a continuous covariate. We visualized the results on regression plots.

Publication bias was assessed by performing the methodology of Deeks et al., including the modified funnel plot [27].

## 3. Results

### 3.1. Search and Selection

Altogether 7649 citations were identified, and after duplicate removal and title–abstract selection, 89 were eligible for full-text screening. Of these, 18 articles [9,14,28,29,30,31,32,33,34,35,36,37,38,39,40,41,42,43], including 1548 patients met the inclusion criteria. The selection process is detailed in Figure S1.

### 3.2. Basic Characteristics and Eligibility Criteria of Included Studies

The included studies were published between January 1992 and September 2022. The number of patients with DMI (≥50% of myometrial infiltration) was 520 (33.6% of the total number of patients). The mean patient age was reported in nine studies, ranging from 54.4 to 69 years, the median age was reported in four further studies, ranging between 54 and 69 years. Most of the included studies were prospective (n = 14). Ten studies were single-center, whereas three had multicenter design. TVS was conducted in nine studies by a single expert examiner, while in six studies, it was performed by multiple examiners. However, MRI was generally interpreted by multiple examiners (n = 10) rather than a single examiner (n = 5). The basic characteristics of the enrolled studies are detailed in Table 1.

The inclusion and exclusion criteria of the included articles are summarized in Table S2. In four studies, TVS was performed by specialists who were aware of the IETA (International Endometrial Tumor Analysis) recommendations [44].

### 3.3. Diagnostic Performance of TVS vs. MRI

**Figure 1:**
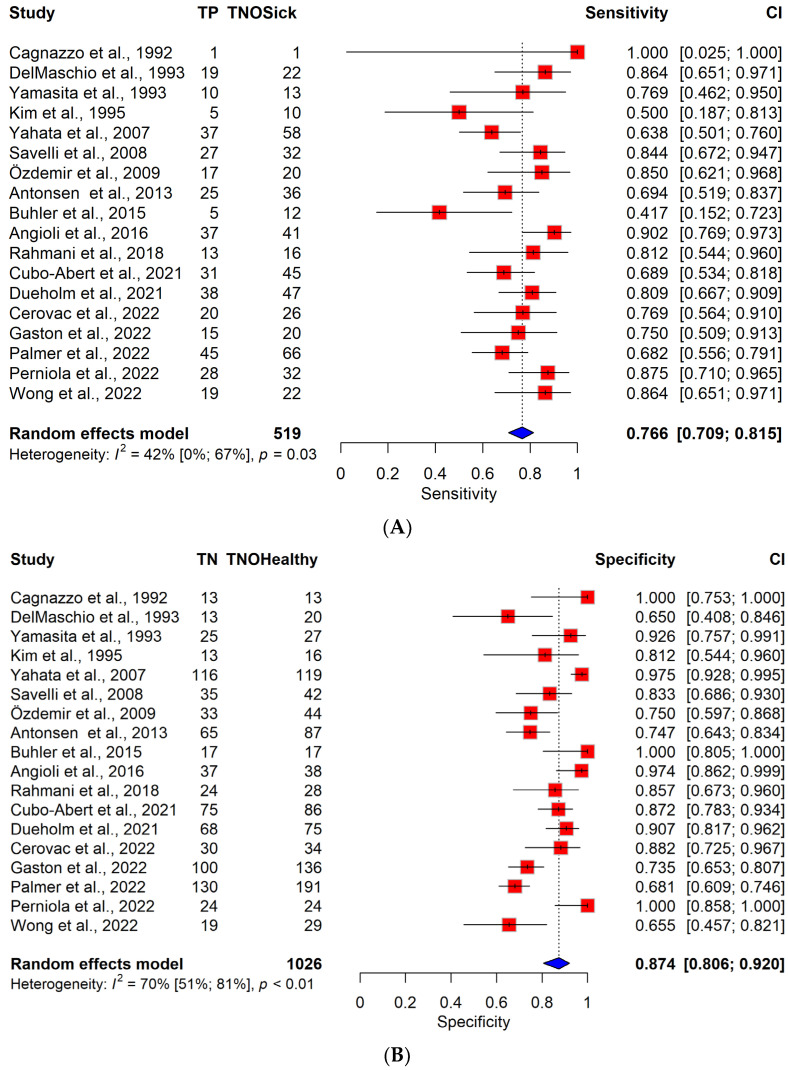

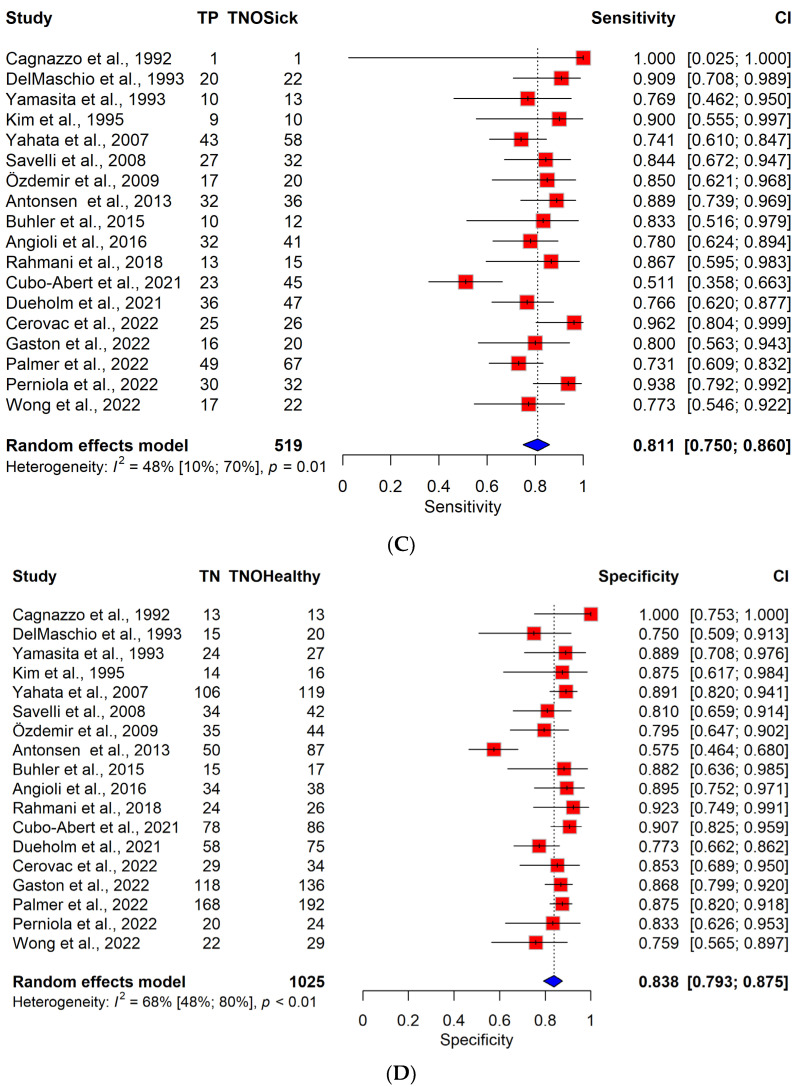
(**A**) Forest plot of TVS for sensitivity across all articles included [9,14,28,29,30,31,32,33,34,35,36,37,38,39,40,41,42,43]. Abbreviations: TP: number of true positive patients. TN: number of true negative patients. TNOHealthy: total number of healthy patients. TNOSick: total number of patients diagnosed with endometrial cancer. CI: confidence interval. TVS: transvaginal sonography. MRI: magnetic resonance imaging. The results of the pooled model are visualized as well. Each red square represents the point estimate of the effect size for a specific study, and the horizontal line through the square indicates the 95% confidence interval (CI). The blue diamond at the bottom represents the overall pooled effect size, with its width representing the 95% CI. Heterogeneity is indicated by I^2^ and *p*-values, representing the variability between the included studies. (**B**) Forest plot of TVS for specificity across all articles included. (**C**) Forest plot of MRI for sensitivity across all articles included. (**D**) Forest plot of MRI for specificity across all articles included.

The pooled sensitivity for DMI sensitivity was 76.6% (95% CI, 70.9–81.4%) for TVS and 81.1% (95% CI, 74.9–85.9%) for MRI, respectively. The pooled specificity was 87.4% (95% CI, 80.6–92%) for TVS and 83.8% (95% CI, 79.2–87.5%) for MRI. The difference between the specificity and sensitivity of the two imaging methods was not significant (sensitivity: *p* = 0.116, specificity: *p* = 0.707). The pooled sensitivity and specificity are presented in ROC plots (Figure S2) and forest plots (Figure 1).

With regard to publications after 2013 (since the last meta-analysis on this topic) [8], the corresponding forest plots and ROC plots are included in Figure S3. No significant difference between the sensitivity and specificity of TVS and MRI was observed (sensitivity: *p* = 0.504, specificity: *p* = 0.843). Changes in sensitivities and specificities over the years are visualized in Figure S4. The *p*-values of the meta-regressions and the regression plots do not indicate any systematic change with time.

### 3.4. Diagnostic Performance of TVS vs. MRI in a Cohort of Patients with Low-Grade Endometrial Cancer

Four studies [33,36,39,41] were included in the low-grade subgroup, and three of these enrolled exclusively low-grade EC patients. This subgroup consisted of 577 patients.

A separate analysis of low-grade EC patients resulted in a sensitivity of 71.9% (95% CI, 64.2–78.4%) for TVS and 70.1% (95% CI, 56.9–80.6%) for MRI, respectively. The difference between these values was not statistically significant (*p* = 0.495). On the other hand, a statistically significant difference (*p* = 0.044) was observed between the specificity of the two groups: a 74.7% (95% CI, 65.3–82.1%) specificity for TVS and an 87.2% (95% CI, 83.1–90.4%) specificity for MRI. Clinically, this result is particularly relevant in patients with low-grade endometrial cancer, where the higher sensitivity of MRI allowed for a more careful assessment of fertility-sparing operations.

**Figure 2:**
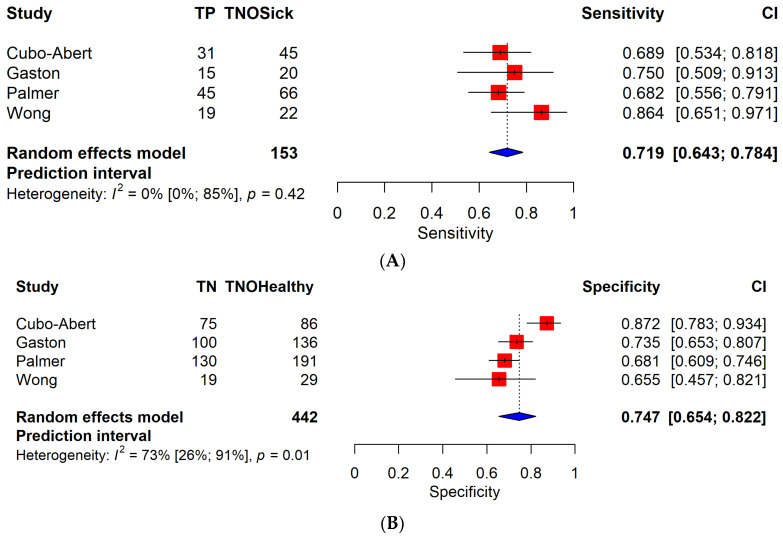

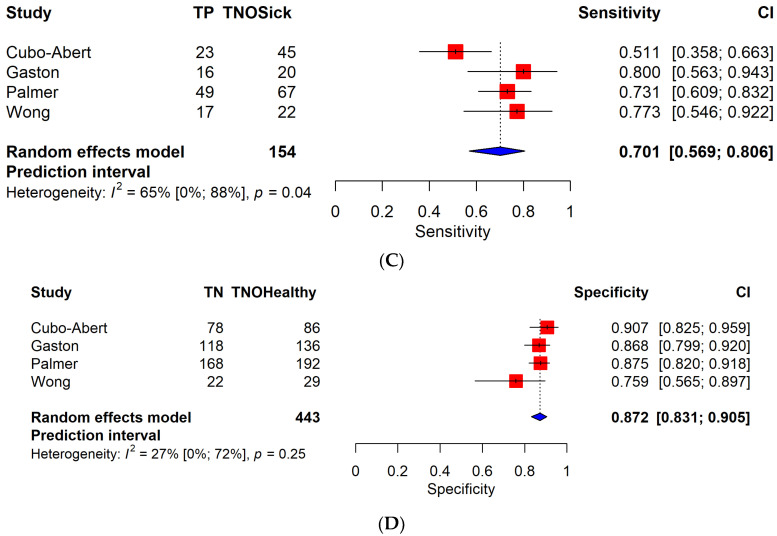
(**A**) Forest plot of TVS for sensitivity; only low-grade EC articles are included [33,36,39,41]. Abbreviations: TP: number of true positive patients. TN: number of true negative patients. TNOHealthy: total number of healthy patients. TNOSick: total number of patients diagnosed with endometrial cancer. CI: confidence interval. TVS: transvaginal sonography. MRI: magnetic resonance imaging. The results of the pooled model are visualized as well. Each red square represents the point estimate of the effect size for a specific study, and the horizontal line through the square indicates the 95% confidence interval (CI). The blue diamond at the bottom represents the overall pooled effect size, with its width representing the 95% CI. Heterogeneity is indicated by I^2^ and *p*-values, representing the variability between the included studies. (**B**) Forest plot of TVS for specificity, with only low-grade EC articles included. (**C**) Forest plot of MRI for sensitivity, with only low-grade EC articles included. (**D**) Forest plot of MRI for specificity, with only low-grade EC articles included.

Figure 2 shows forest plots for TVS and MRI for low-grade EC. ROC plots are shown in Figure S5.

### 3.5. MRI Performance According to the Sequences Used

Seventeen articles provided data on the MRI sequences used [14,28,29,30,31,32,33,34,35,36,37,38,39,40,41,42,43]. We divided these articles into two groups: the first group included studies that used exclusively T1 and/or T2 sequences (T1–T2 group), the second group included studies that used DCE and/or DWI (DCE-DWI group), in addition to T1 and T2 sequences. No significant difference was observed between the sensitivity and specificity of the two methods (sensitivity: *p* = 0.755, specificity: *p* = 0.788). Forest plots and ROC plots are presented in Figure S6.

### 3.6. No Myometrial Invasion vs. Invasion of Any Depth

In five articles, including 329 patients [14,31,34,42,43], it was possible to compare TVS and MRI for no myometrial invasion vs. myometrial invasion of any depth. The pooled sensitivity for the detection of myometrial invasion was 89.9% (95% CI, 76.8–95.9%) for TVS and 91.1% (95% CI, 78.4–96.6%) for MRI, respectively. The pooled specificity was 42.6% (95% CI, 30.9–55.1%) for TVS and 46.1% (95% CI, 35.6–56.9%) for MRI. No significant difference was observed (sensitivity: *p* = 0.911, specificity: *p* = 0.145). Forest plots and ROC plots are shown in Figure S7.

### 3.7. Risk of Bias Assessment

For the domain Patient Selection, most of the articles clearly defined inclusion criteria. The overall risk of bias was moderate to low. Articles excluding high-grade EC patients were not considered to be at a high risk of bias [33,36,39].

For the index test domain, we performed separate assessments of TVS and MRI. Most of the studies were considered to be at a low risk of bias.

In three studies [29,38,39], the exact method adopted to assess DMI with the TVS or the infiltration depth was not clearly defined, leaving the risk of bias unclear. In one study [30], none of the previous criteria were well defined, and the study was therefore considered to be at a high risk of bias.

For MRI examination, most of the studies were considered to have low risk of bias. In seven articles [14,28,29,30,38,39,40], either the exact methodology or a clear description of how DMI was assessed were missing. These studies were considered to be at an unclear risk of bias.

For the reference test domain, four studies [34,38,41,43], clearly reported that the pathologist performing the final histopathology was blinded to the results of the imaging procedures. These were considered to be at a low risk of bias.

For the flow and timing domain, the overall risk of bias was low to moderate. Eleven studies clearly identified the time between the index test and surgery; therefore, these were considered to be at a low risk of bias. In the rest of the studies, these data were missing, making the risk of bias unclear.

The results of the risk of bias assessment are presented in Table S3. A detailed description of the risk of bias applied is described in Section S2.

In terms of applicability, patient selection and reference standard domains were considered to have low risk of bias across all articles.

In terms of the clinical importance of the low-grade subgroup, a separate risk of bias assessment was performed for this subgroup, which is presented in Section S3.

### 3.8. Publication Bias and Heterogeneity

On the basis of the funnel plot, no evidence of serious publication bias could be observed for the diagnostic accuracy of the two methods (TVS: *p* = 0.214, MRI: *p* = 0.052) (Figure S8).

Moderate heterogeneity was observable for sensitivity (TVS: I^2^ = 42% [0–67%], MRI: I^2^ = 48% [10–70%]) for all-grade EC patients. Specificity was moderate for both imaging methods (TVS: I^2^ = 70% [51–81%], MRI: I^2^ = 68% [48–80%]).

## 4. Discussion

In our study, we found no significant difference between the diagnostic performance of TVS and MRI when both low- and high-grade EC patients were included. On the other hand, when only low-grade cases were analyzed, the specificity of MRI proved to be significantly higher. This might highlight the potential benefits of MRI, interpreted by expert examiners, in patients with low-grade EC, when TVS has limited diagnostic abilities due to different factors affecting visibility.

In 2017, Alcazar et al. published a meta-analysis on the assessment of DMI, including studies on 560 patients, published before 2013 [8]. They concluded that MRI showed superior sensitivity compared to TVS in detecting DMI in women with EC. However, the difference between the two imaging modalities was not statistically significant. The elaboration of studies conducted after 2013 showed a slightly better TVS sensitivity and specificity, as well as MRI specificity. However, the pooled sensitivity of MRI was reduced.

Ultrasound imaging has advantages, such as being more accessible, repeatable and time-efficient, and it can be performed without the use of a contrasting agent. However, disadvantages include the fact that it is operator-dependent, and accuracy is affected by tumor size, tumor vascularization density, tumor vessel architecture and histological grading [45,46]. In a recent meta-analysis by Tameish et al., conducted exclusively on patients with low-grade EC, neither sensitivity nor specificity was significant between TVS and MRI in the assessment of DMI [47]. However, in our subgroup analysis on the same articles, we observed a significantly better specificity of MRI. This difference can be attributed to the different data used in our study. In one article, two different radiologists evaluated MRI examinations [39]. We included data from more experienced examiners, in agreement with the recommendations of the ESGO/ESTRO/ESP guidelines [4].

To improve the diagnostic accuracy of TVS, IETA studies were conducted on the application of terminology in relation to tumor stage, grade and histological type [44], high-risk EC prediction [6] and ultrasound-based prognostic models [48,49]. A histological feature, microcystic elongated and fragmented (MELF) pattern is associated with lymph node metastases and an advanced tumor stage in EC; however, it does not affect preoperative ultrasound staging and does not increase the risk of underestimating DMI in preoperative ultrasound staging [50]. The diagnostic accuracy of TVS in patients with concomitant benign uterine pathologies is often limited [51].

The addition of clinical data and radiomics to ultrasound can result in promising models, able to discriminate between different EC risk groups. In 2022, Moro et al. demonstrated that radiomics exhibits a capacity to differentiate low-risk endometrial cancer from other forms and demonstrates superior discrimination between high-risk endometrial cancer and alternative types. Nevertheless, the integration of radiomics features into clinical ultrasound models did not yield a significant enhancement in overall performance [52]. Radiomics applied to ultrasound images and machine learning models demonstrated promising performance in other female genital tumors, such as ovarian and myometrial lesions [53,54].

Spagnol et al. reported comparable diagnostic accuracy of 3D-TVS to MRI for the detection of DMI [55]. In this meta-analysis, which includes five studies, with a total of 450 patients, the pooled sensitivity was 77% (95% CI, 66–85%) for 3D-TVS and 80% (95% CI, 73–86%) for MRI, with a specificity of 74% vs. 83% for detecting DMI. In our study, the pooled sensitivity was similar to these data, and TVS specificity was better. In a recent meta-analysis by Ziogas et al., no significant difference was observed between 2D-TVS and 3D-TVS in terms of sensitivity and specificity (80.4% vs. 77.6% and 82.8% vs. 81.6%, respectively, *p* = 0.960 and *p* = 0.733) [13]. Perniola et al. reported that tumor volume estimation on 2D-TVS, 3D-TVS and MRI showed correlations among all three methods, indicating that 2D-TVS can be sufficient for myometrial infiltration assessment [14]. However, Costas et al. conducted a meta-analysis on the predictive value of 3D-TVS in DMI assessment, which resulted in a pooled sensitivity of 84% (95% CI, 73–90%) and a specificity of 82% (95% CI, 75–88%). These findings were superior to the outcomes of our investigation [56].

TVS interpretation methods (subjective or different objective modalities) were different in our included studies. The IETA 4 study found that, in terms of sensitivity, subjective assessment provided comparable results to objective methods (Karlsson’s method, minimal tumor-free margin) in the hands of experienced ultrasound examiners [6]. Subjective assessment was the best method to predict DMI, especially in patients with low-grade EC. Another study concluded that the subjective ultrasound evaluation of DMI performed better than objective methods in most measurements, although statistically significant improvements were observed only in terms of sensitivity [57].

MRI, the other imaging technique of interest, provides detailed information on soft tissues. However, MRI images are more expensive to obtain, are not tolerated by many patients due to claustrophobia, are time consuming, are not suitable for patients who are very obese or have metal implants, foreign bodies or impaired kidney functions [58,59]. In contrast to TVS, MRI is considered as less reliant on operator skills. Nevertheless, a multicenter network study suggests the use of the European Society of Urogenital Radiology (ESUR) guidelines [60]. Different MRI sequences were used in the articles included in our meta-analysis, which might have influenced the sensitivity and specificity obtained in the studies. In a recent meta-analysis, Bi et al. concluded that diagnostic accuracy is highest using T2-weighted imaging, dynamic contrast-enhanced MRI, and diffusion-weighted imaging for the detection of DMI [61]. The pooled sensitivity and specificity were 79% (95% CI, 75–83%) and 81% (95% CI, 78–83%), respectively. Similar results were obtained in our study for articles on patients with low- and high-grade EC. Other studies found no significant difference in the diagnostic performance between DWI and DCE for the diagnosis of DMI in EC [62,63]. Radiomics applied to MRI and artificial intelligence are valuable tools that can aid clinicians in the proper diagnosis and management of EC [64]. These tools have shown promising results in other gynecological tumors as well [65,66,67].

Our study highlighted the role of TVS in the preoperative assessment of DMI. Considering the greater number of patients enrolled compared to previous meta-analyses and the improvement of imaging methods over a decade, we found that 2D-TVS maintains its reliability in the preoperative evaluation of EC. TVS is cheaper and more widely available than MRI, which can be particularly important in low-resource countries.

Regarding the strengths of our analysis, we followed our protocol, registered in advance. Further strengths were the rigorous methodology, meticulous analysis of data, several subgroup analyses, such as a low-grade only patient group. MRI and TVS were performed on the same cohort of patients, which allowed a better comparison of the two methods. Finally, the studies generally showed a moderate-to-good quality in terms of the index test and reference standard definition.

The generalization of our results is problematic for low-grade tumors, due to the low number of patients enrolled and the barely significant difference between the specificity of TVS and MRI. For a non-expert examiner, this difference was not statistically significant. Different interpretations of the imaging data were applied in the articles. The presence of moderate and high risks of bias in some of the domains is an additional limitation.

Heterogeneity might be attributed to different scanning and interpretation methods, as well as to the different proportions of EC types in the study populations. In addition, operator experience might also play a role.

On the basis of previous evidence, there are clear benefits of rapidly integrating results into clinical practice [68].

Our results suggest that TVS can be a good alternative imaging modality when MRI is not an option. TVS is cheaper and more easily available. The results of our subgroups should be interpreted with caution, as further prospective data collection would be needed to assess the diagnostic accuracy of TVS and MRI in the same patient cohort with low-grade EC.

## 5. Conclusions

In conclusion, our study showed that ultrasound maintains its diagnostic performance in the detection of deep myometrial infiltration in endometrial cancer. For all-grade tumors, TVS showed higher sensitivity, whereas MRI had higher specificity. Further research should be conducted on the performance of TVS and MRI to assess the presence or absence of myometrial infiltration and to provide valuable information, especially in fertility-sparing wishes.

Furthermore, there is a need to explore how artificial intelligence and radiomics can improve the diagnostic performance and predictive values of ultrasound and MRI. External validation of prognostic models would also be reasonable.

## Figures and Tables

**Table 1:** Basic characteristics of the included articles.

Author	Study Design	Study Period	Number of Centers	Number of Patients	Number of DMI (≥50% Myometrial Infiltration)	Pathologist Blinded to Imaging	TVS Observer	MRI Observer
Cubo-Abert, 2021 [33]	prospective	October 2013–July 2018	single	131	45	NA	single	multiple
Gaston, 2022 [36]	prospective	January 2016–March 2020	single	156	20	yes	single	single
Palmer, 2023 [39]	prospective	January 2017–June 2019	multi	259	67	NA	multiple	multiple
Wong, 2022 [41]	prospective	October 2015–October 2018	single	51	22	yes	single	single
Perniola, 2022 [14]	prospective	March 2019–March 2021	single	56	32	NA	single	single
Cerovac, 2022 [32]	prospective	July 2019–April 2021	single	60	26	NA	single	single
Rahmani, 2018 [40]	prospective	October 2009–December 2012	single	45	16	NA	single	single
Dueholm, 2021 [35]	prospective	November 2013–December 2015	single	122	47	NA	multiple	multiple
Cagnazzo, 1992 [31]	NA	NA	NA	14	1	NA	NA	NA
DelMaschio, 1993 [34]	prospective	NA	NA	42	22	yes	single	multiple
Yamasita, 1993 [43]	prospective	NA	NA	40	13	yes	single	multiple
Kim, 1995 [37]	prospective	January 1991–April 1994	NA	26	10	NA	multiple	multiple
Yahata, 2007 [42]	retrospective	January 1995–April 2004	NA	177	58	NA	NA	NA
Savelli, 2008 [9]	prospective	February 2000–May 2004	multi	74	32	NA	multiple	multiple
Özdemir, 2009 [38]	prospective	January 2007–November2008	single	64	20	NA	single	multiple
Antonsen, 2013 [29]	prospective	September 2009–January 2012	multi	123	36	NA	multiple	multiple
Buhler, 2015 [30]	retrospective	July 2012–July 2014	single	29	12	NA	multiple	multiple
Angioli, 2016 [28]	prospective	January 2012–February 2015	single	79	41	NA	NA	NA

NA: no data available, DMI: deep myometrial invasion.

## Data Availability

The datasets used in this study can be found in the full-text articles that were included in the systematic review and meta-analysis.

## Supplementary Materials

The following supporting information can be downloaded at: https://www.mdpi.com/article/10.3390/cancers16050907/s1, Section S1: search key. Section S2: risk of bias assessment methodology. Section S3: risk of bias assessment for low-grade EC subgroup. Table S1: PRISMA 2020 checklist. Table S2: eligibility criteria in each included article. Table S3: risk of bias (quality assessment of diagnostic accuracy studies 2). Figure S1: PRISMA flow chart. Figure S2: pooled sensitivity and specificity of TVS (A) and MRI (B). Figure S3: articles with data published since the last meta-analysis on this topic. Figure S4: regression plots of sensitivity and specificity over the years. Figure S5: pooled sensitivity and specificity of TVS (A) and MRI (B) in low-grade EC patients. Figure S6: MRI data grouped by the sequences used. Figure S7: no myometrial invasion vs. myometrial invasion. Figure S8: funnel plots, all articles included for TVS (A) and MRI (B).

## References

[1] Sung H., Ferlay J., Siegel R.L., Laversanne M., Soerjomataram I., Jemal A., Bray F. (2021). Global Cancer Statistics 2020: GLOBOCAN Estimates of Incidence and Mortality Worldwide for 36 Cancers in 185 Countries. CA Cancer J. Clin..

[2] Gu B., Shang X., Yan M., Li X., Wang W., Wang Q., Zhang C. (2021). Variations in incidence and mortality rates of endometrial cancer at the global, regional, and national levels, 1990–2019. Gynecol. Oncol..

[3] Åkesson Å., Wolmesjö N., Adok C., Milsom I., Dahm-Kähler P. (2021). Lymphadenectomy, obesity and open surgery are associated with surgical complications in endometrial cancer. Eur. J. Surg. Oncol..

[4] Concin N., Matias-Guiu X., Vergote I., Cibula D., Mirza M.R., Marnitz S., Ledermann J., Bosse T., Chargari C., Fagotti A. (2021). ESGO/ESTRO/ESP guidelines for the management of patients with endometrial carcinoma. Int. J. Gynecol. Cancer.

[5] Epstein E., Van Holsbeke C., Mascilini F., Måsbäck A., Kannisto P., Ameye L., Fischerova D., Zannoni G., Vellone V., Timmerman D. (2011). Gray-scale and color Doppler ultrasound characteristics of endometrial cancer in relation to stage, grade and tumor size. Ultrasound Obstet. Gynecol..

[6] Verbakel J.Y., Mascilini F., Wynants L., Fischerova D., Testa A.C., Franchi D., Frühauf F., Cibula D., Lindqvist P.G., Fruscio R. (2020). Validation of ultrasound strategies to assess tumor extension and to predict high-risk endometrial cancer in women from the prospective IETA (International Endometrial Tumor Analysis)-4 cohort. Ultrasound Obstet. Gynecol..

[7] Restaino S., Paglietti C., Arcieri M., Biasioli A., Della Martina M., Mariuzzi L., Andreetta C., Titone F., Bogani G., Raimondo D. (2023). Management of Patients Diagnosed with Endometrial Cancer: Comparison of Guidelines. Cancers.

[8] Alcázar J.L., Gastón B., Navarro B., Salas R., Aranda J., Guerriero S. (2017). Transvaginal ultrasound versus magnetic resonance imaging for preoperative assessment of myometrial infiltration in patients with endometrial cancer: A systematic review and meta-analysis. J. Gynecol. Oncol..

[9] Savelli L., Ceccarini M., Ludovisi M., Fruscella E., De Iaco P.A., Salizzoni E., Mabrouk M., Manfredi R., Testa A.C., Ferrandina G. (2008). Preoperative local staging of endometrial cancer: Transvaginal sonography vs. magnetic resonance imaging. Ultrasound Obstet. Gynecol..

[10] Savelli L., Testa A.C., Mabrouk M., Zannoni L., Ludovisi M., Seracchioli R., Scambia G., De Iaco P. (2012). A prospective blinded comparison of the accuracy of transvaginal sonography and frozen section in the assessment of myometrial invasion in endometrial cancer. Gynecol. Oncol..

[11] Kumar S., Bandyopadhyay S., Semaan A., Shah J.P., Mahdi H., Morris R., Munkarah A., Ali-Fehmi R. (2011). The role of frozen section in surgical staging of low risk endometrial cancer. PLoS ONE.

[12] Green R.W., Valentin L., Alcazar J.L., Chiappa V., Erdodi B., Franchi D., Frühauf F., Fruscio R., Guerriero S., Graupera B. (2018). Endometrial cancer off-line staging using two-dimensional transvaginal ultrasound and three-dimensional volume contrast imaging: Intermethod agreement, interrater reliability and diagnostic accuracy. Gynecol. Oncol..

[13] Ziogas A., Xydias E., Kalantzi S., Papageorgouli D., Liasidi P.N., Lamari I., Daponte A. (2022). The diagnostic accuracy of 3D ultrasound compared to 2D ultrasound and MRI in the assessment of deep myometrial invasion in endometrial cancer patients: A systematic review. Taiwan J. Obstet. Gynecol..

[14] Perniola G., Derme M., Manganaro L., Satta S., Palaia I., Di Donato V., Muzii L., Panici P.B. (2022). Correlation between preoperative imaging biomarkers and histological prognostic factors in endometrial cancer: A prospective study. J. Clin. Ultrasound.

[15] Matsuo K., Mandelbaum R.S., Matsuzaki S., Klar M., Roman L.D., Wright J.D. (2021). Ovarian conservation for young women with early-stage, low-grade endometrial cancer: A 2-step schema. Am. J. Obstet. Gynecol..

[16] Rodolakis A., Scambia G., Planchamp F., Acien M., Di Spiezio Sardo A., Farrugia M., Grynberg M., Pakiz M., Pavlakis K., Vermeulen N. (2023). ESGO/ESHRE/ESGE Guidelines for the fertility-sparing treatment of patients with endometrial carcinoma. Int. J. Gynecol. Cancer.

[17] Page M.J., McKenzie J.E., Bossuyt P.M., Boutron I., Hoffmann T.C., Mulrow C.D., Shamseer L., Tetzlaff J.M., Akl E.A., Brennan S.E. (2021). The PRISMA 2020 statement: An updated guideline for reporting systematic reviews. BMJ.

[18] Cumpston M., Li T., Page M.J., Chandler J., Welch V.A., Higgins J.P., Thomas J. (2019). Updated guidance for trusted systematic reviews: A new edition of the Cochrane Handbook for Systematic Reviews of Interventions. Cochrane Database Syst. Rev..

[19] McHugh M.L. (2012). Interrater reliability: The kappa statistic. Biochem. Med..

[20] Ouzzani M., Hammady H., Fedorowicz Z., Elmagarmid A. (2016). Rayyan-a web and mobile app for systematic reviews. Syst. Rev..

[21] Freeman S.C., Kerby C.R., Patel A., Cooper N.J., Quinn T., Sutton A.J. (2019). Development of an interactive web-based tool to conduct and interrogate meta-analysis of diagnostic test accuracy studies: MetaDTA. BMC Med. Res. Methodol..

[22] Reitsma J.B., Glas A.S., Rutjes A.W., Scholten R.J., Bossuyt P.M., Zwinderman A.H. (2005). Bivariate analysis of sensitivity and specificity produces informative summary measures in diagnostic reviews. J. Clin. Epidemiol..

[23] Chu H., Cole S.R. (2006). Bivariate meta-analysis of sensitivity and specificity with sparse data: A generalized linear mixed model approach. J. Clin. Epidemiol..

[24] Burke D.L., Ensor J., Snell K.I.E., van der Windt D., Riley R.D. (2018). Guidance for deriving and presenting percentage study weights in meta-analysis of test accuracy studies. Res. Synth. Methods.

[25] Stijnen T., Hamza T.H., Ozdemir P. (2010). Random effects meta-analysis of event outcome in the framework of the generalized linear mixed model with applications in sparse data. Stat. Med..

[26] Pustejovsky J.E., Tipton E. (2022). Meta-analysis with Robust Variance Estimation: Expanding the Range of Working Models. Prev. Sci..

[27] Deeks J.J., Macaskill P., Irwig L. (2005). The performance of tests of publication bias and other sample size effects in systematic reviews of diagnostic test accuracy was assessed. J. Clin. Epidemiol..

[28] Angioli R., Plotti F., Capriglione S., Scaletta G., Dugo N., Aloisi A., Piccolo C.L., Del Vescovo R., Terranova C., Zobel B.B. (2016). Preoperative local staging of endometrial cancer: The challenge of imaging techniques and serum biomarkers. Arch. Gynecol. Obstet..

[29] Antonsen S.L., Jensen L.N., Loft A., Berthelsen A.K., Costa J., Tabor A., Qvist I., Hansen M.R., Fisker R., Andersen E.S. (2013). MRI, PET/CT and ultrasound in the preoperative staging of endometrial cancer—A multicenter prospective comparative study. Gynecol. Oncol..

[30] Buhler J., Routiot T., Polet-Lefebvre K., Morel O. (2015). Feedback of ultrasound and RMI in the staging of endometrial carcinoma in early stage. Gynecol. Obstet. Fertil..

[31] Cagnazzo G., D’Addario V., Martinelli G., Lastilla G. (1992). Depth of myometrial invasion in endometrial cancer: Preoperative assessment by transvaginal ultrasonography and magnetic resonance imaging. Ultrasound Obstet. Gynecol..

[32] Cerovac A., Ljuca D., Arnautalic L., Habek D., Bogdanovic G., Mustedanagic-Mujanovic J., Grgic G. (2022). Efficacy of transvaginal ultrasound versus magnetic resonance imaging for preoperative assessment of myometrial invasion in patients with endometrioid endometrial cancer: A prospective comparative study. Radiol. Oncol..

[33] Cubo-Abert M., Díaz-Feijoo B., Bradbury M., Rodríguez-Mías N.L., Vera M., Pérez-Hoyos S., Gómez-Cabeza J.J., Gil-Moreno A. (2021). Diagnostic performance of transvaginal ultrasound and magnetic resonance imaging for preoperative evaluation of low-grade endometrioid endometrial carcinoma: Prospective comparative study. Ultrasound Obstet. Gynecol..

[34] DelMaschio A., Vanzulli A., Sironi S., Spagnolo D., Belloni C., Garancini P., Taccagni G.L. (1993). Estimating the depth of myometrial involvement by endometrial carcinoma: Efficacy of transvaginal sonography vs MR imaging. AJR Am. J. Roentgenol..

[35] Dueholm M., Hjorth I.M., Dahl K., Marinovskij E., Ørtoft G. (2021). Preoperative prediction of high-risk endometrial cancer by expert and non-expert transvaginal ultrasonography, magnetic resonance imaging, and endometrial histology. Eur. J. Obstet. Gynecol. Reprod. Biol..

[36] Gastón B., Muruzábal J.C., Lapeña S., Modroño A., Guarch R., García de Eulate I., Alcázar J.L. (2022). Transvaginal Ultrasound Versus Magnetic Resonance Imaging for Assessing Myometrial Infiltration in Endometrioid Low Grade Endometrial Cancer: A Prospective Study. J. Ultrasound Med..

[37] Kim S.H., Kim H.D., Song Y.S., Kang S.B., Lee H.P. (1995). Detection of deep myometrial invasion in endometrial carcinoma: Comparison of transvaginal ultrasound, CT, and MRI. J. Comput. Assist. Tomogr..

[38] Özdemir S., Çelik C., Emlik D., Kiresi D., Esen H. (2009). Assessment of myometrial invasion in endometrial cancer by transvaginal sonography, doppler ultrasonography, magnetic resonance imaging and frozen section. Int. J. Gynecol. Cancer.

[39] Palmér M., Åkesson Å., Marcickiewicz J., Blank E., Hogström L., Torle M., Mateoiu C., Dahm-Kähler P., Leonhardt H. (2023). Accuracy of transvaginal ultrasound versus MRI in the PreOperative Diagnostics of low-grade Endometrial Cancer (PODEC) study: A prospective multicentre study. Clin. Radiol..

[40] Rahmani M., Heydari S., Mousavi A., Ahmadinejad N., Azhdeh S., Shakiba M. (2018). Accuracy of imaging in preoperative local staging of endometrial cancer: Could imaging predict low risk patients?. Int. J. Women’s Health Reprod. Sci..

[41] Wong M., Amin T., Thanatsis N., Naftalin J., Jurkovic D. (2022). A prospective comparison of the diagnostic accuracies of ultrasound and magnetic resonance imaging in preoperative staging of endometrial cancer. J. Gynecol. Oncol..

[42] Yahata T., Aoki Y., Tanaka K. (2007). Prediction of myometrial invasion in patients with endometrial carcinoma: Comparison of magnetic resonance imaging, transvaginal ultrasonography, and gross visual inspection. Eur. J. Gynaecol. Oncol..

[43] Yamashita Y., Mizutani H., Torashima M., Takahashi M., Miyazaki K., Okamura H., Ushijima H., Ohtake H., Tokunaga T. (1993). Assessment of myometrial invasion by endometrial carcinoma: Transvaginal sonography vs contrast-enhanced MR imaging. AJR Am. J. Roentgenol..

[44] Epstein E., Fischerova D., Valentin L., Testa A.C., Franchi D., Sladkevicius P., Frühauf F., Lindqvist P.G., Mascilini F., Fruscio R. (2018). Ultrasound characteristics of endometrial cancer as defined by International Endometrial Tumor Analysis (IETA) consensus nomenclature: Prospective multicenter study. Ultrasound Obstet. Gynecol..

[45] Alcazar J.L., Pineda L., Martinez-Astorquiza Corral T., Orozco R., Utrilla-Layna J., Juez L., Jurado M. (2015). Transvaginal/transrectal ultrasound for assessing myometrial invasion in endometrial cancer: A comparison of six different approaches. J. Gynecol. Oncol..

[46] Fischerova D., Frühauf F., Zikan M., Pinkavova I., Kocián R., Dundr P., Nemejcova K., Dusek L., Cibula D. (2014). Factors affecting sonographic preoperative local staging of endometrial cancer. Ultrasound Obstet. Gynecol..

[47] Tameish S., Florez N., Vidal J.R.P., Chen H., Vara J., Alcázar J.L. (2023). Transvaginal ultrasound versus magnetic resonance imaging for preoperative assessment of myometrial infiltration in patients with low-grade endometrioid endometrial cancer: A systematic review and head-to-head meta-analysis. J. Clin. Ultrasound.

[48] Wynants L., Verbakel J.Y.J., Valentin L., De Cock B., Pascual M.A., Leone F.P.G., Sladkevicius P., Heremans R., Alcazar J.L., Votino A. (2022). The Risk of Endometrial Malignancy and Other Endometrial Pathology in Women with Abnormal Uterine Bleeding: An Ultrasound-Based Model Development Study by the IETA Group. Gynecol. Obstet. Investig..

[49] Van Holsbeke C., Ameye L., Testa A.C., Mascilini F., Lindqvist P., Fischerova D., Frühauf F., Fransis S., de Jonge E., Timmerman D. (2014). Development and external validation of new ultrasound-based mathematical models for preoperative prediction of high-risk endometrial cancer. Ultrasound Obstet. Gynecol..

[50] Eriksson L.S.E., Nastic D., Frühauf F., Fischerova D., Nemejcova K., Bono F., Franchi D., Fruscio R., Ghioni M., Haak L.A. (2019). Clinical and Ultrasound Characteristics of the Microcystic Elongated and Fragmented (MELF) Pattern in Endometrial Cancer According to the International Endometrial Tumor Analysis (IETA) criteria. Int. J. Gynecol. Cancer.

[51] Capozzi V.A., Merisio C., Rolla M., Pugliese M., Morganelli G., Cianciolo A., Gambino G., Armano G., Sozzi G., Riccò M. (2021). Confounding factors of transvaginal ultrasound accuracy in endometrial cancer. J. Obstet. Gynaecol..

[52] Moro F., Albanese M., Boldrini L., Chiappa V., Lenkowicz J., Bertolina F., Mascilini F., Moroni R., Gambacorta M.A., Raspagliesi F. (2022). Developing and validating ultrasound-based radiomics models for predicting high-risk endometrial cancer. Ultrasound Obstet. Gynecol..

[53] Chiappa V., Bogani G., Interlenghi M., Salvatore C., Bertolina F., Sarpietro G., Signorelli M., Castiglioni I., Raspagliesi F. (2021). The Adoption of Radiomics and machine learning improves the diagnostic processes of women with Ovarian MAsses (the AROMA pilot study). J. Ultrasound.

[54] Chiappa V., Interlenghi M., Salvatore C., Bertolina F., Bogani G., Ditto A., Martinelli F., Castiglioni I., Raspagliesi F. (2021). Using rADioMIcs and machine learning with ultrasonography for the differential diagnosis of myometRiAL tumors (the ADMIRAL pilot study). Radiomics and differential diagnosis of myometrial tumors. Gynecol. Oncol..

[55] Spagnol G., Noventa M., Bonaldo G., Marchetti M., Vitagliano A., Laganà A.S., Cavallin F., Scioscia M., Saccardi C., Tozzi R. (2022). Three-dimensional transvaginal ultrasound vs magnetic resonance imaging for preoperative staging of deep myometrial and cervical invasion in patients with endometrial cancer: Systematic review and meta-analysis. Ultrasound Obstet. Gynecol..

[56] Costas T., Belda R., Alcazar J.L. (2022). Transvaginal three-dimensional ultrasound for preoperative assessment of myometrial invasion in patients with endometrial cancer: A systematic review and meta-analysis. Med. Ultrason..

[57] Frühauf F., Zikan M., Semeradova I., Dundr P., Nemejcova K., Dusek L., Cibula D., Fischerova D. (2017). The Diagnostic Accuracy of Ultrasound in Assessment of Myometrial Invasion in Endometrial Cancer: Subjective Assessment versus Objective Techniques. BioMed Res. Int..

[58] Sammet S. (2016). Magnetic resonance safety. Abdom. Radiol..

[59] Murphy K.J., Brunberg J.A. (1997). Adult claustrophobia, anxiety and sedation in MRI. Magn. Reson. Imaging.

[60] Soneji N.D., Bharwani N., Ferri A., Stewart V., Rockall A. (2018). Pre-operative MRI staging of endometrial cancer in a multicentre cancer network: Can we match single centre study results?. Eur. Radiol..

[61] Bi Q., Chen Y., Wu K., Wang J., Zhao Y., Wang B., Du J. (2020). The Diagnostic Value of MRI for Preoperative Staging in Patients with Endometrial Cancer: A Meta-Analysis. Acad. Radiol..

[62] Wang L.J., Tseng Y.J., Wee N.K., Low J.J.H., Tan C.H. (2021). Diffusion-weighted imaging versus dynamic contrast-enhanced imaging for pre-operative diagnosis of deep myometrial invasion in endometrial cancer: A meta-analysis. Clin. Imaging.

[63] Andreano A., Rechichi G., Rebora P., Sironi S., Valsecchi M.G., Galimberti S. (2014). MR diffusion imaging for preoperative staging of myometrial invasion in patients with endometrial cancer: A systematic review and meta-analysis. Eur. Radiol..

[64] Di Donato V., Kontopantelis E., Cuccu I., Sgamba L., Golia D’Augè T., Pernazza A., Della Rocca C., Manganaro L., Catalano C., Perniola G. (2023). Magnetic resonance imaging-radiomics in endometrial cancer: A systematic review and meta-analysis. Int. J. Gynecol. Cancer.

[65] Chiappa V., Bogani G., Interlenghi M., Vittori Antisari G., Salvatore C., Zanchi L., Ludovisi M., Leone Roberti Maggiore U., Calareso G., Haeusler E. (2023). Using Radiomics and Machine Learning Applied to MRI to Predict Response to Neoadjuvant Chemotherapy in Locally Advanced Cervical Cancer. Diagnostics.

[66] Shrestha P., Poudyal B., Yadollahi S., Wright D.E., Gregory A.V., Warner J.D., Korfiatis P., Green I.C., Rassier S.L., Mariani A. (2022). A systematic review on the use of artificial intelligence in gynecologic imaging—Background, state of the art, and future directions. Gynecol. Oncol..

[67] Pesapane F., De Marco P., Rapino A., Lombardo E., Nicosia L., Tantrige P., Rotili A., Bozzini A.C., Penco S., Dominelli V. (2023). How Radiomics Can Improve Breast Cancer Diagnosis and Treatment. J. Clin. Med..

[68] Hegyi P., Erőss B., Izbéki F., Párniczky A., Szentesi A. (2021). Accelerating the translational medicine cycle: The Academia Europaea pilot. Nat. Med..

