# Supplementary material for: Diagnostic Accuracy of Transvaginal Ultrasound and Magnetic Resonance Imaging for the Detection of Myometrial Infiltration in Endometrial Cancer: A Systematic Review and Meta-Analysis
Source: Cancers (Basel). 2024 Feb 23;16(5):907. doi: 10.3390/cancers16050907 (PMC10931203; doi:10.3390/cancers16050907)

# Supplementary Material

## Title

Diagnostic Accuracy of Transvaginal Ultrasound and Magnetic Resonance Imaging for the Detection of Myometrial Infiltration in Endometrial Cancer: a Systematic review and Meta-analysis


## Authors

István Madár[1,2], Anett Szabó[1,3], Gábor Vleskó[1,2], Péter Hegyi[1,4,5], Nándor Ács[1,2], Péter Fehérvári[1,6], Tamás Kói[1,7], Emma Kálovics[1], Gábor Szabó[1,2]

## Affiliations:

1. Centre for Translational Medicine, Semmelweis University, 1088 Budapest, Hungary.
2. Department of Obstetrics and Gynecology, Semmelweis University, 1088 Budapest, Hungary
3. Department of Urology, Semmelweis University, 1082 Budapest, Hungary
4. Institute for Translational Medicine, Medical School, University of Pécs, 7624 Pécs, Hungary
5. Institute of Pancreatic Diseases, Semmelweis University, 1083 Budapest, Hungary
6. Department of Biostatistics, University of Veterinary Medicine, 1078 Budapest, Hungary
7. Stochastics Department, Budapest University of Technology and Economics, 1111 Budapest, Hungary

## Corresponding author

Gábor Szabó MD, PhD

Postal address: H-1088 Budapest, Baross utca 27, Hungary

Tel.: +36 20 4554710

E-mail address: szabo.gabor6@med.semmelweis-univ.hu

**TABLE OF CONTENT**

**Section S1. S**earch key

**Section S2.** Risk of bias assessment methodology

**Section S3.** Risk of bias assessment for low-grade EC subgroup

**Table S1.** PRISMA 2020 checklist

**Table S2.** Eligibility criteria in each included article

**Table S3.** Risk of Bias (Quality Assessment of Diagnostic Accuracy Studies 2)

**Figure S1.** PRISMA Flow chart

**Figure S2.** Pooled sensitivity and specificity of TVS and MRI

**Figure S3.** Articles with data published since the last meta-analysis on this topic

**Figure S4.** Regression plots of sensitivity and specificity over the years

**Figure S5.** Pooled sensitivity and specificity of TVS and MRI in low-grade EC patients

**Figure S6.** MRI data grouped by the sequences used

**Figure S7.** No myometrial invasion vs. myometrial invasion

**Figure S8.** Funnel plots, all articles included for TVS (A) and MRI (B)

**Section S1.** Search key

(endometr* OR uter* OR womb OR UCEC) AND (neoplasm* OR tumor OR tumour OR carcinoma OR cancer* OR malign* OR UCEC) AND (ultraso* OR sonograph* OR TVS) AND (magnetic resonance imaging OR MRI)

**Section S2.** Risk of bias assessment methodology

QUADAS-2 is structured so that the 4 key domains are each rated in terms of the risk of bias and the concern regarding applicability to the research question (as defined above). Each key domain has a set of signaling questions to help reach the judgments regarding bias and applicability.

Overall ratings for each domain were assigned as carrying 'low' (green), 'unclear' (yellow) or 'high' (red) risk of bias, based on the items included in each domain.

**Domain 1:**

**1. Risk of bias concerning patient selection:** (1) Low risk of bias was attributed if authors enrolled consecutive or a random sample of patients, if a case-control design was avoided and if inappropriate exclusions were avoided. Inclusion of patients exclusively of low-grade endometrial cancer was not considered source of bias, as most of the studies enrolled patients mostly affected by low-grade endometrial cancer. (2) Unclear bias was attributed if authors did not specify the abovementioned inclusion criteria (3) High risk of bias was attributed to articles where authors enrolled other than consecutive or random samples of patients, in case-control designs and if patients were inappropriately excluded. Thus, retrospective studies without clearly indicating the patient selection methods were considered high risk of bias. In case the patients were prescreened

by trainees with less experienced and afterwards enrolled in the study, we considered it to be of high risk of bias. Studies excluding patients with “unequivocal”, “inconclusive” or “unclear” ultrasound or magnetic resonance imaging results were considered as high risk of bias. Further inclusion in this risk group was the case when the study design (prospective/retrospective) was not clear.

**2. Concerns regarding applicability:** (1) Low risk of bias was attributed to articles where there was no concern that the included patients matched the review question. (2) Unclear risk of bias was attributed if authors did not specify the abovementioned criteria. Based on our inclusion criteria, only studies of low risk of bias regarding this domain were included.

**Domain 2:**

**1. Risk of bias concerning index test (TVS):** (1) Low risk of bias was attributed if the index test results were interpreted without knowledge of the results of the reference standard. Further criterium was a description of a pre-specified threshold. In our study this meant a description of the deep myometrial infiltration assessment and interpretation. (2) Unclear risk of bias was attributed if either assessment or interpretation was not properly described. (3) High risk of bias was attributed if none of the abovementioned criteria were fulfilled. If additional methods (eg. 3D ultrasound) were used for a better visualization, high risk of bias was attributed.

**2. Concerns regarding applicability (TVS**): (1) Low risk of bias was attributed if there was no concern that the index test, its implementation, or interpretation differed from the review question. (2) High risk of bias was attributed if the implementation of the index test differed from the review question.

**Domain 3:**

**1. Risk of bias concerning index test (MRI):** (1) Low risk of bias was attributed if the index test results were interpreted without knowledge of the results of the reference standard. Further criterium was a description of a pre-specified threshold. In our study this meant a description of the deep myometrial infiltration assessment and interpretation. (2) Unclear risk of bias was attributed if either assessment or interpretation was not properly described. (3) High risk of bias was attributed if none of the abovementioned criteria were not fulfilled.

**2. Concerns regarding applicability (MRI):** (1) Low risk of bias was attributed if there was no concern that the index test, its implementation, or interpretation differed from the review question. (2) high risk of bias was attributed if the implementation of index test differed from the review question.

**Domain 4:**

**1. Risk of bias concerning reference standard:** in our study the reference standard was the definitive histology obtained from the hysterectomy. Currently, the gold standard method for confirmation of deep myometrial infiltration is the abovementioned method. Being a compulsory inclusion criterion, in all our studies the reference standard was likely to correctly classify the target condition. (1) Low risk of bias was attributed if the pathologist evaluating the definitive hysterectomy specimen was blinded to the result of the imaging methods. (2) Unclear risk of bias was attributed if no data about the abovementioned criterium was available. (3) High risk was attributed if the pathologist was certainly aware of the imaging results.

**2. Concerns regarding applicability of the reference standard:** Since exclusively articles with the abovementioned reference standard were included, no concerns about the target condition

matching the review question arise. Thus, at this point, all our articles were considered to have low risk of bias.

**Domain 5:**

**1. Risk of bias concerning flow and timing:** (1) Low risk of bias was attributed if all patients received the reference standard, if they all received the same reference standard, and if all of them were included in the analysis. Another inclusion criterion was an appropriate interval between the index test and reference test. If there was less than 40 days between the index test and reference test, we considered it of low risk. (2) Unclear risk of bias was attributed if no data on the abovementioned interval was available. (3) High risk of bias was attributed if more than 40 days passed between the index test and reference test.

**Section S3.** Risk of bias assessment for low-grade EC subgroup

For the domain patient selection, all studies were considered to have low risk of bias. Regarding the index tests TVS and MRI, three out of four studies (Cubo-Abert et al., Gaston et al., Wong et al.) clearly stated the methodology and the interpretation of the methods, thus these were considered to have low risk of bias. However, in one study (Palmer et al), due to the insufficient data on this aspect, unclear risk of bias was attributed to both index test domains. Only one study (Wong et al.) stated clearly that pathologist was blinded to the imagistic results, this way being considered to have low risk of bias. In the other studies, no information on this aspect was provided, thus these were considered to have unclear risk of bias.

Two studies (Cubo-Abert et al., Wong et al.) stated clearly the time elapsed between the imaging methods and operation. These were considered to have low risk of bias for the domain “Flow and

timing". The other two studies (Gaston et al., Palmer et al.) did not provided data on this domain, so they were considered to have unclear risk of bias.

Regarding the applicability, all studies had low risk of bias for the "Patient selection" and "Reference standard" domains. In one article (Cubo-Abert et al.) the authors used real-time three-dimensional ultrasound in special cases, this was considered high risk of bias at the applicability of index test TVS. Due to unclear data, in one study (Palmer et al.) both imaging methods had intermediate risk of bias at the applicability. Two articles (Gaston et al., Wong et al.) had low risk of bias for both index tests.

**Table S1.** PRISMA 2020 checklist

| Section and topic | Item # | Checklist item | Location where item is reported |
|---|---|---|---|
| **Title** | | | |
| Title | 1 | Identify the report as a systematic review. | 2 |
| **Abstract** | | | |
| Abstract | 2 | See the PRISMA 2020 for Abstracts checklist | 2 |
| **Introduction** | | | |
| Rationale | 3 | Describe the rationale for the review in the context of existing knowledge. | 2 |
| Objectives | 4 | Provide an explicit statement of the objective(s) or question(s) the review addresses. | 2-3 |
| **Methods** | | | |
| Eligibility criteria | 5 | Specify the inclusion and exclusion criteria for the review and how studies were grouped for the syntheses. | 3 |
| Information sources | 6 | Specify all databases, registers, websites, organizations, reference lists and other sources searched or consulted to identify studies. Specify the date when each source was last searched or consulted. | 3 |
| Search strategy | 7 | Present the full search strategies for all databases, registers, and websites, including any filters and limits used. | 3 |
| Selection process | 8 | Specify the methods used to decide whether a study met the inclusion criteria of the review, including how many reviewers screened each record and each report retrieved, whether they worked independently, and if applicable, details of automation tools used in the process. | 3-4 |
| Data collection process | 9 | Specify the methods used to collect data from reports, including how many reviewers collected data from each report, whether they worked independently, any processes for obtaining or confirming data from study investigators, and if applicable, details of automation tools used in the process. | 3-4 |
| Data items | 10a | List and define all outcomes for which data were sought. Specify whether all results that were compatible with each outcome domain in each study were sought (e.g., for all measures, time points, analyses), and if not, the methods used to decide which results to collect. | 3-4 |
| | 10b | List and define all other variables for which data were sought (e.g., participant and intervention characteristics, funding sources). Describe any assumptions made about any missing or unclear information. | 3-4 |
| Study risk of bias assessment | 11 | Specify the methods used to assess risk of bias in the included studies, including details of the tool(s) used, how many reviewers assessed each study and whether they worked independently, and if applicable, details of automation tools used in the process. | 9-10 |
| Effect measures | 12 | Specify for each outcome the effect measure(s) (e.g., risk ratio, mean difference) used in the synthesis or presentation of results. | 3-4 |
| Synthesis methods | 13a | Describe the processes used to decide which studies were eligible for each synthesis (e.g., tabulating the study intervention characteristics and comparing against the planned groups for each synthesis). | 4 |
| | 13b | Describe any methods required to prepare the data for presentation or synthesis, such as handling of missing summary statistics, or data conversions. | 3-4 |
| | 13c | Describe any methods used to tabulate or visually display results of individual studies and syntheses. | 3-4 |
| | 13d | Describe any methods used to synthesize results and provide a rationale for the choice(s). If meta-analysis was performed, describe the model(s), method(s) to identify the presence and extent of statistical heterogeneity, and software package(s) used. | 3-4 |
| | 13e | Describe any methods used to explore possible causes of heterogeneity among study results (e.g. subgroup analysis, meta-regression). | 3-4 |
| | 13f | Describe any sensitivity analyses conducted to assess robustness of the synthesized results. | 3-4 |
| Reporting bias assessment | 14 | Describe any methods used to assess risk of bias due to missing results in a synthesis (arising from reporting biases). | 3 |

| | | | |
|---|---|---|---|
| Certainty assessment | 15 | Describe any methods used to assess certainty (or confidence) in the body of evidence for an outcome. | 3-4 |
| **Results** | | | |
| Study selection | 16a | Describe the results of the search and selection process, from the number of records identified in the search to the number of studies included in the review, ideally using a flow diagram. | SM Figure S1 |
| | 16b | Cite studies that might appear to meet the inclusion criteria, but which were excluded, and explain why they were excluded. | - |
| Study characteristics | 17 | Cite each included study and present its characteristics. (see Table 1. ) | Table 1 |
| Risk of bias in studies | 18 | Present assessments of risk of bias for each included study. (see Table S3) | SM Table S3 |
| Results of individual studies | 19 | For all outcomes, present, for each study: (a) summary statistics for each group (where appropriate) and (b) an effect estimate and its precision (e.g. confidence/credible interval), ideally using structured tables or plots. | 6-9 |
| Results of syntheses | 20a | For each synthesis, briefly summarize the characteristics and risk of bias among contributing studies. | 9-10 |
| | 20b | Present results of all statistical syntheses conducted. If meta-analysis was done, present for each the summary estimate and its precision (e.g. confidence/credible interval) and measures of statistical heterogeneity. If comparing groups, describe the direction of the effect. | 6-9 |
| | 20c | Present results of all investigations of possible causes of heterogeneity among study results. | 10 |
| | 20d | Present results of all sensitivity analyses conducted to assess the robustness of the synthesized results. | 10 |
| Reporting biases | 21 | Present assessments of risk of bias due to missing results (arising from reporting biases) for each synthesis assessed. | 10 |
| Certainty of evidence | 22 | Present assessments of certainty (or confidence) in the body of evidence for each outcome assessed. | 6-9 |
| **Discussion** | | | |
| Discussion | 23a | Provide a general interpretation of the results in the context of other evidence. | 10-12 |
| | 23b | Discuss any limitations of the evidence included in the review. | 10-12 |
| | 23c | Discuss any limitations of the review processes used. | 10-12 |
| | 23d | Discuss implications of the results for practice, policy, and future research. | 10-12 |
| **Other information** | | | |
| Registration and protocol | 24a | Provide registration information for the review, including register name and registration number, or state that the review was not registered. | 2 |
| | 24b | Indicate where the review protocol can be accessed, or state that a protocol was not prepared. | 2-3 |
| | 24c | Describe and explain any amendments to information provided at registration or in the protocol. | 2-3 |
| Support | 25 | Describe sources of financial or non-financial support for the review, and the role of the funders or sponsors in the review. | 12 |
| Competing interests | 26 | Declare any competing interests of review authors. | 13 |
| Availability of data, code, and other materials | 27 | Report which of the following are publicly available and where they can be found: template data collection forms; data extracted from included studies; data used for all analyses; analytic code; any other materials used in the review. | SM |

SM: Supplementary Material

**Table S2.** Eligibility criteria in each included article

| Author (year) | Inclusion criteria ("verbatim") | Exclusion criteria ("verbatim") |
|---|---|---|
| Angioli et al (2016) | Inclusion criteria for enrollment were as follows: (1) age between 18 and 80 years; (2) Eastern Cooperative Oncology Group performance status 0–2 according to World Health Organization criteria; (3) informed consent obtained from the patients. | Exclusion criteria included: (1) abnormal cardiac, hematological, renal, respiratory, and/or hepatic functions; (2) presence of a secondary malignancy; (3) concomitant benign and/or malignant adnexal pathologies, (4) claustrophobia, (5) any mental illness.<br>Patients were included in the study only if both physicians, performing TVS and MRI, could give an unequivocal opinion on myometrial and cervical infiltration by the neoplasm. |
| Antonsen et al. (2016) | Patients with a histological diagnosis of EC or atypical endometrial hyperplasia (AEH) were consecutively invited to participate in the Danish endometrial cancer study (ENDOMET).<br>Patients with premalignant cancers, cured skin cancer of non-melanoma type and former breast cancer were included. | (1) claustrophobia, severe obesity or difficulties in co-operation; (2) severe kidney-disease that contraindicated intravenous contrast-agents; and (3) additional malignant disease, current or former. (4) Patients with certain implanted magnetic objects were excluded from MRI. |
| Buhler et al. (2015) | endometrial cancer stage I, who underwent a pelvic ultrasound and MRI for the assessment of myometrial infiltration. | NA |
| Cagnazzo et al. (1992) | Thirty patients, aged between 46 and 78 years, diagnosed as having FIGO I endometrial carcinoma on the basis of D&C, were included in the study. | NA |
| Cerovac et al. (2022) | 60 women with a histopathological proven endometrioid EC by dilatation and curettage | Women with another malignant disease, who previously have surgery for EC or other malignant disease, who have previously received chemotherapy and/or radiotherapy due to a malignant disease, women with a histopathological proven EC who preoperatively have made pelvic CT, cases that were diagnosed incidentally after hysterectomy. |
| Cubo-Abert et al. (2021) | All patients diagnosed with EC on pipelle biopsy or hysteroscopic-directed biopsy prior to surgery were considered potentially eligible for inclusion. | Exclusion criteria included Grade-3 EEC or non-endometrioid EC on preoperative biopsy, contraindication for surgical treatment and/or cases in which it was not possible to evaluate the endometrium by TVS and/or MRI |
| DelMaschio et al. (1993) | Fifty-one consecutive patients with histologically proved endometrial carcinoma were considered for inclusion in this prospective study. | Nine patients in whom pathologic examination of the surgical specimen showed the presence of advanced disease were excluded. |
| Dueholm et al. (2021) | This prospective cohort study included women with a diagnosis of endometrial cancer or atypical hyperplasia (n = 266) based on the final hysterectomy specimen. | Exclusion criteria for hysteroscopy and expert-TVS were: Serious comorbidity excluding adjuvant chemotherapy (n = 12) and inducing operative risk, Tumors not considered suitable for hysteroscopy (n = 29), e.g. unfavorable tumors on preoperative office sampling (non-endometrioid or grade 3 endometrioid tumors), giving theoretical risk of spread of high-risk tumor type. Tumors suspected of having myometrial invasion to the serosa on non-expert-TVS, inducing risk of perforation. MRI (n = 175) was subsequently performed: When there was no absolute confidence regarding myometrial involvement (MI) <50 % on non-expert-TVS, when an |

| | | |
|---|---|---|
| | | unfavorable tumor type was present, or cancer FIGO stage 2–4 was suspected (n = 158). MRI was also performed (n = 31) when there was no available tumor type, and hysteroscopy was planned in relation to MRI. MRI was, however, not performed in women unable to undergo MRI because of serious obesity, comorbidity, or claustrophobia (n = 14) |
| Gaston et al. (2022) | Consecutive cases with a diagnosis of well (G1) or moderately differentiated (G2) endometrioid carcinoma after preoperative endometrial sampling obtained either by blind aspiration or hysteroscopy were considered as eligible candidates for this study. | A preoperative biopsy result of high-risk endometrial cancer (poorly differentiated endometrioid carcinoma or non-endometrioid histology) and not being suitable for TVS or MRI were considered as exclusion criteria for this study. |
| Kim et al. (1995) | This study included 26 women with histologically proven and endometrial carcinoma in whom TVUS, CT, and MRI were performed and who underwent surgical exploration during the period between January 1991 and April 1994. | NA |
| Özdemir et al. (2009) | Patients were included in the study only if both physicians, performing TVS and MRI, could give an unequivocal opinion on myometrial invasion by the neoplasm. | The patients were excluded from the study because of inconclusive sonographic results or unclear findings at MRI. |
| Palmer et al. (2023) | All patients during the time of the study presenting with biopsy-verified low-grade EC (FIGO Grade 1-2) without apparent extra-uterine manifestations and planned for primary surgical treatment were considered for inclusion. | Patients with contradictions to MRI such as severe claustrophobia; patients with estimated glomerular filtration rate <30 ml/min or allergy to gadolinium contrast medium3; patients with body mass index (BMI) exceeding 45 kg/m2 who possibly would not fit in the MRI gantry; women <18 years of age or with ongoing pregnancy; non-proficient in Swedish or not cognitively able to understand the study protocol. Three patients were included despite having a BMI >45 kg/m2 as the high BMI was not noted until after successfully completing the MRI examination. |
| Perniola et al. (2022) | Inclusion criteria were as follows: age ≥18 years, written informed consent, biopsy-proven endometrial carcinoma, and absence of previous neoadjuvant chemo- or radiotherapy treatment. | The exclusion criteria were advanced disease (FIGO stage III and IV), patients with other coexisting malignant tumors. |
| Rahmani et al. (2018) | Inclusion criteria were diagnosis of endometrial carcinoma with endometrial curettage or biopsy. | Patients who had never had sexual intercourse, patients who were not candidates for surgery due to advanced disease stage or poor clinical condition, and patients with contraindications for using MRI or using gadolinium during MRI were excluded. |
| Savelli et al. (2008) | Patients were included in the study only if both physicians, performing TVS and MRI, could give an unequivocal opinion on myometrial and cervical infiltration by the neoplasm. | Twelve patients evaluated during the study period were excluded from the database because of inconclusive sonographic results (seven cases, 8%) or unclear findings at MRI (five cases, 6%) owing to the presence of several uterine myomas and adenomyosis. Two patients were excluded because of uncertainty in the histological diagnosis with regard to the true extent of myometrial infiltration by the neoplasm.<br><br>We decided to exclude from the analysis those patients in whom a high level of uncertainty |

| | | |
|---|---|---|
| | | limited the preoperative TVS- or MRI-based staging, or the definitive pathological staging. |
| Wong et al. (2022) | We included women with a history of postmenopausal bleeding or unscheduled vaginal bleeding while on hormone replacement therapy (HRT).<br>Only endometrial cancers with epithelial or mixed epithelial and mesenchymal histological types were included, i.e., endometrioid, mucinous, serous, clear cell, mixed, undifferentiated and carcinosarcoma.<br>In our final statistical analysis, we included only women who underwent both ultrasound and MRI examinations. | We excluded women who did not undergo hysterectomy following the imaging tests.<br>144 were excluded as the endometrium could not be satisfactorily assessed.<br>Later, we excluded 5 women who had no evidence of malignancy on endometrial biopsy and hysteroscopy.<br>A further 5 women were also excluded as they did not undergo MRI due to claustrophobia, presence of a cardiac pacemaker or morbid obesity. And finally, 7 more women were excluded as they did not undergo hysterectomy due to significant medical co-morbidities or patient moved abroad. |
| Yahata et al. (2007) | One hundred and seventy-seven patients with histopathological diagnoses of endometrial cancer were referred for MRI and TVUS examination between January 1995 and April 2004. | NA |
| Yamashita et al. (1993) | Forty patients 34-80 years old (mean, 58 years) with histologically proved early-stage endometrial carcinoma (primary tumor confined to uterine corpus) were included in the study. | NA |

NA: not available

**Table S3.** Risk of Bias (Quality Assessment of Diagnostic Accuracy Studies 2)

| First author | Publication year | Risk of bias | | | | | Applicability concerns | | | |
|---|---|---|---|---|---|---|---|---|---|---|
| | | Patient selection | Index test TVS | Index test MRI | Reference standard | Flow and timing | Patient selection | Index test TVS | Index test MRI | Reference standard |
| Cubo-Abert et al. | 2021 | ☺ | ☺ | ☺ | 😐 | ☺ | ☺ | ☹ | ☺ | ☺ |
| Gaston et al. | 2022 | ☺ | ☺ | ☺ | 😐 | 😐 | ☺ | ☺ | ☺ | ☺ |
| Palmer et al. | 2023 | ☺ | 😐 | 😐 | 😐 | 😐 | ☺ | 😐 | 😐 | ☺ |
| Wong et al. | 2022 | ☺ | ☺ | ☺ | ☺ | ☺ | ☺ | ☺ | ☺ | ☺ |
| Perniola et al. | 2022 | ☺ | ☺ | 😐 | 😐 | 😐 | ☺ | ☺ | ☺ | ☺ |
| Cerovac et al. | 2022 | ☺ | ☺ | ☺ | 😐 | ☺ | ☺ | ☺ | ☺ | ☺ |
| Rahmani et al. | 2018 | ☺ | ☺ | 😐 | 😐 | 😐 | ☺ | ☺ | 😐 | ☺ |
| Dueholm et al. | 2021 | ☹ | ☺ | ☺ | 😐 | 😐 | ☺ | ☺ | ☺ | ☺ |
| Cagnazzo et al. | 1992 | ☹ | ☺ | ☺ | 😐 | ☺ | ☺ | ☺ | ☺ | ☺ |
| DelMaschio et al. | 1993 | ☺ | ☺ | ☺ | ☺ | ☺ | ☺ | ☺ | ☺ | ☺ |
| Yamashita et al. | 1993 | ☺ | ☺ | ☺ | ☺ | ☺ | ☺ | ☺ | ☺ | ☺ |
| Kim et al. | 1995 | ☺ | ☺ | ☺ | 😐 | ☺ | ☺ | ☺ | ☺ | ☺ |
| Yahata et al. | 2007 | ☹ | ☺ | ☺ | 😐 | ☺ | ☺ | ☺ | ☺ | ☺ |
| Savelli et al. | 2008 | ☹ | ☺ | ☺ | 😐 | 😐 | ☺ | ☺ | ☺ | ☺ |
| Özdemir et al. | 2009 | ☹ | 😐 | 😐 | ☺ | ☺ | ☺ | 😐 | 😐 | ☺ |
| Antonsen et al. | 2013 | ☺ | 😐 | 😐 | 😐 | ☺ | ☺ | 😐 | 😐 | ☺ |
| Buhler et al. | 2015 | ☹ | ☹ | 😐 | 😐 | 😐 | ☺ | ☺ | 😐 | ☺ |
| Angioli et al. | 2016 | ☹ | ☺ | 😐 | 😐 | ☺ | ☺ | ☺ | 😐 | ☺ |

**Figure S1.** PRISMA Flow chart

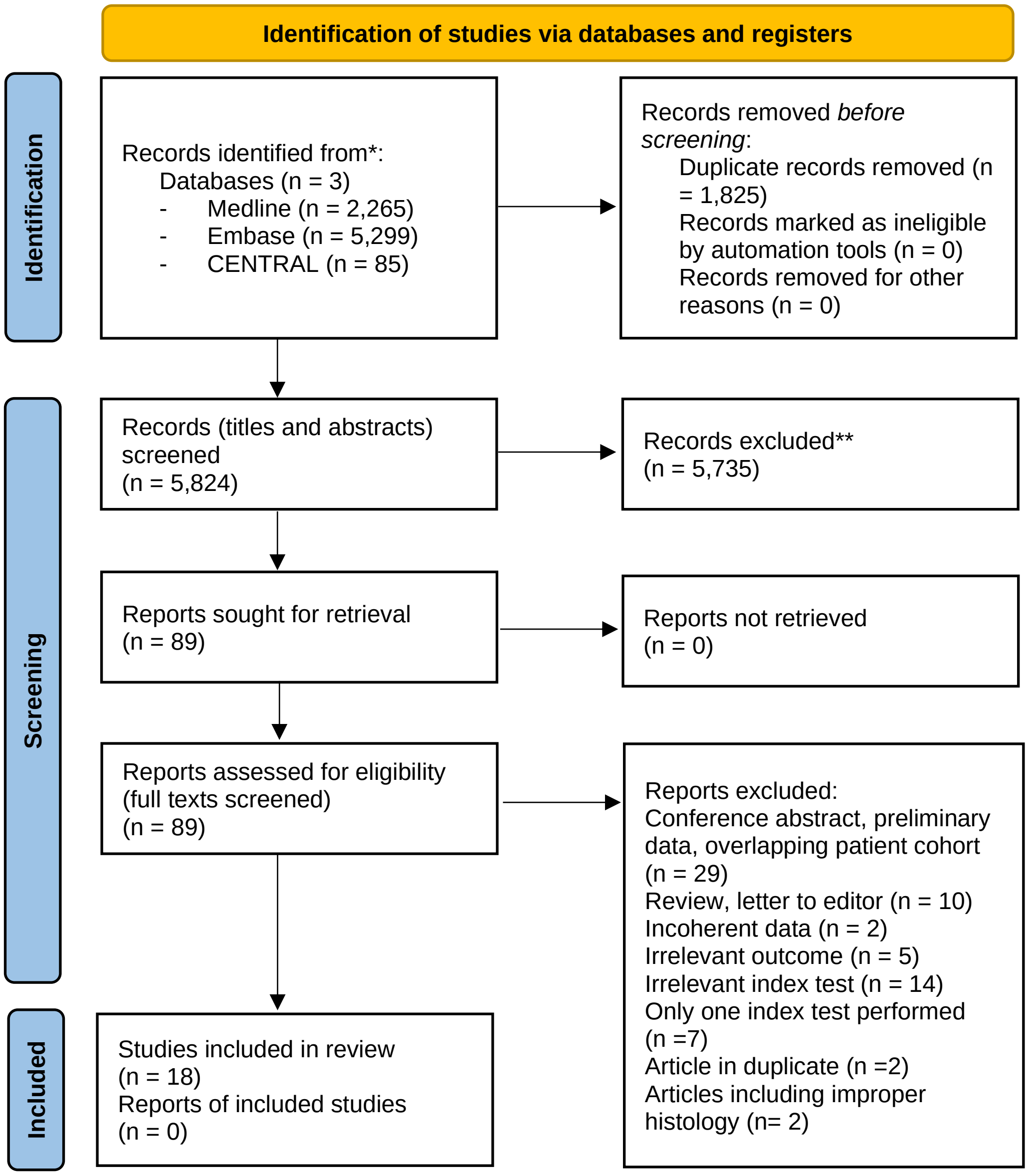


*Consider, if feasible to do so, reporting the number of records identified from each database or register searched (rather than the total number across all databases/registers).

**If automation tools were used, indicate how many records were excluded by a human and how many were excluded by automation tools.

*From:* Page MJ, McKenzie JE, Bossuyt PM, Boutron I, Hoffmann TC, Mulrow CD, et al. The PRISMA 2020 statement: an updated guideline for reporting systematic reviews. BMJ 2021;372:n71. doi: 10.1136/bmj.n71

For more information, visit: http://www.prisma-statement.org/

**Figure S2.** Pooled sensitivity and specificity of transvaginal ultrasound and magnetic resonance imaging in all-grades endometrial cancer patients.

Figures S2.A. Pooled sensitivity and specificity of TVS

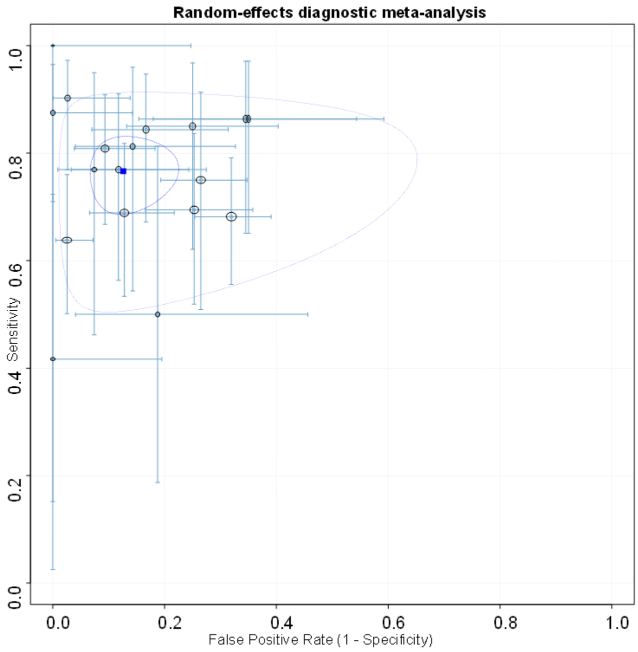


Figure S2. B. Pooled sensitivity and specificity of MRI

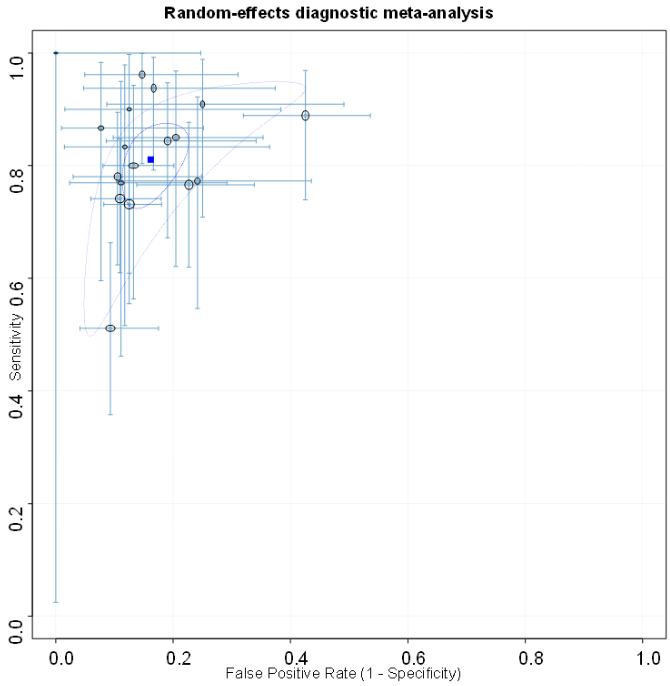

**Figure S3.** Articles with data since the last meta-analysis on this topic. The previous meta-analysis (Alcazar et al., 2017) contained articles issued before 2013.

**Figure S3.A. Forest plot containing the data on TVS sensitivity**

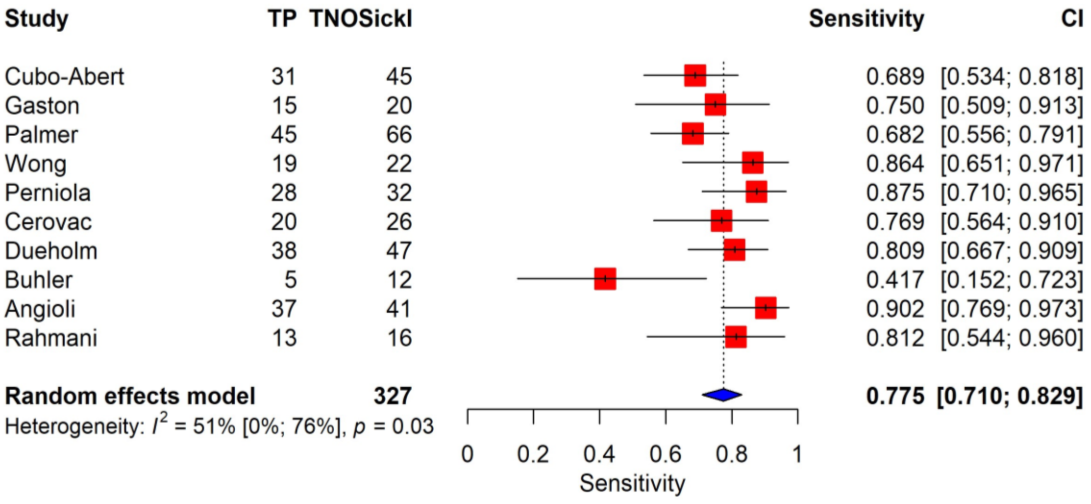


**Figure S3.B. Forest plot containing the data on TVS specificity**

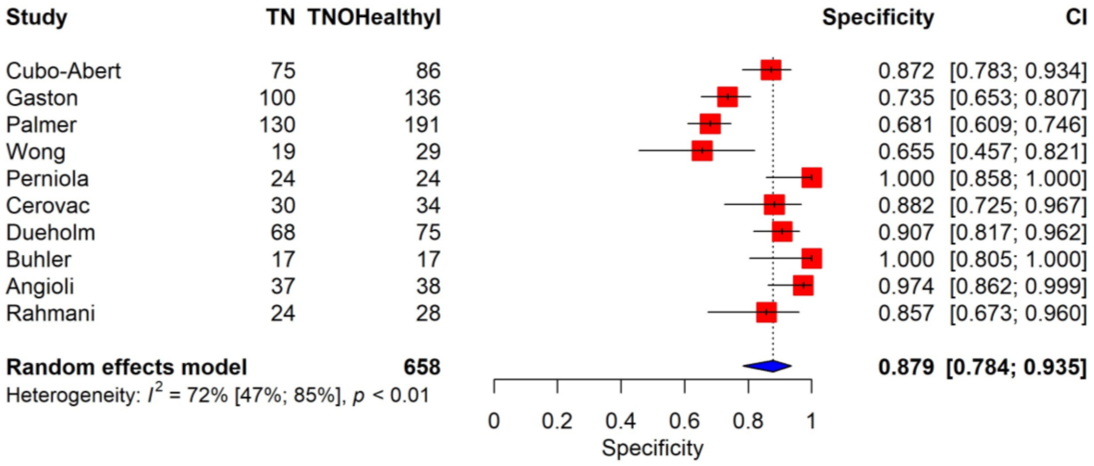


**Figure S3.C. Forest plot containing the data on MRI sensitivity**

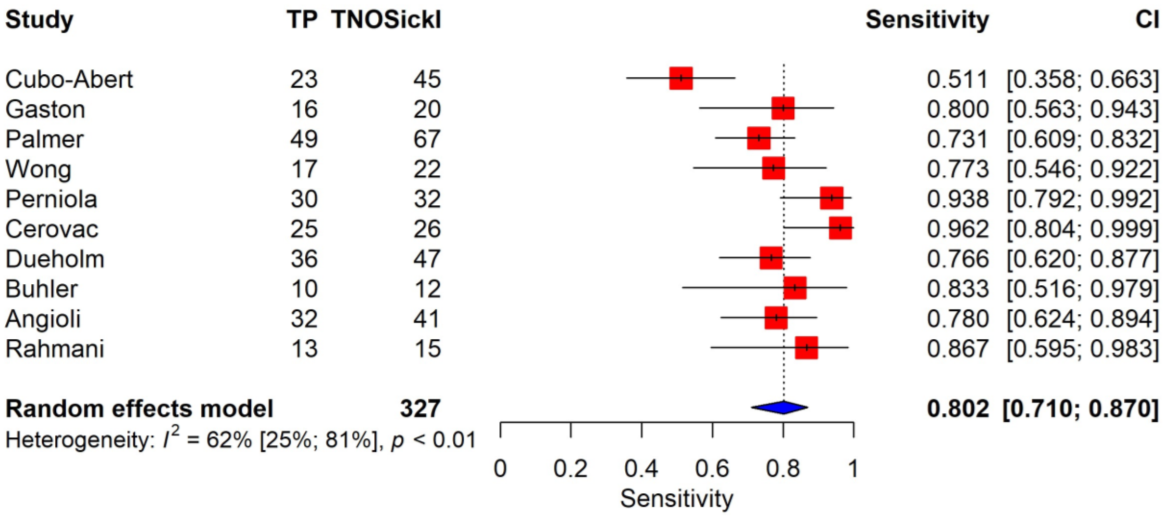

**Figure S3.D. Forest plot containing the data on MRI specificity**

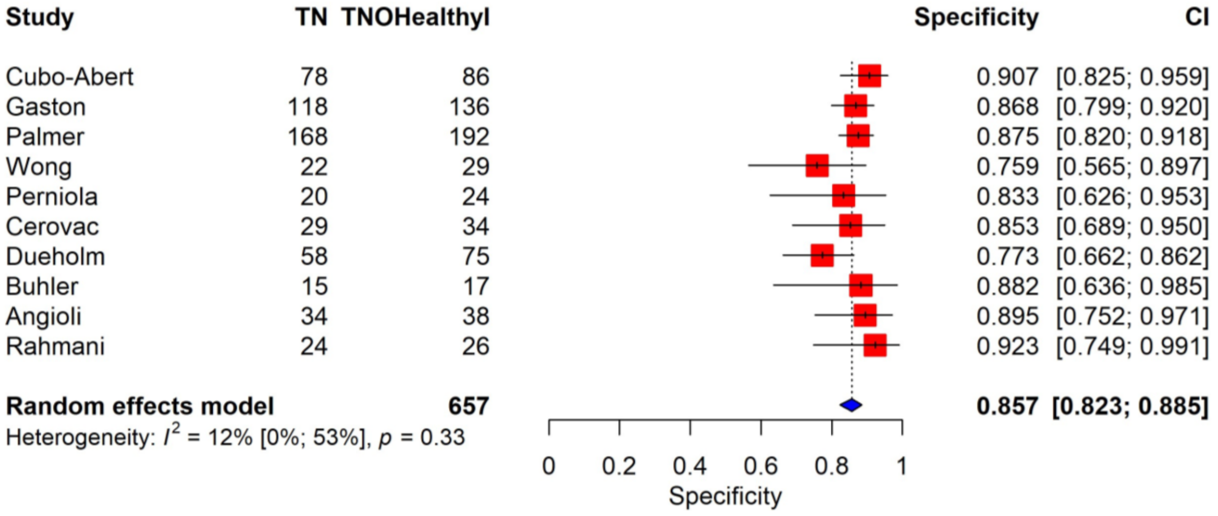


**Figure S3.E. Pooled sensitivity and specificity of TVS**

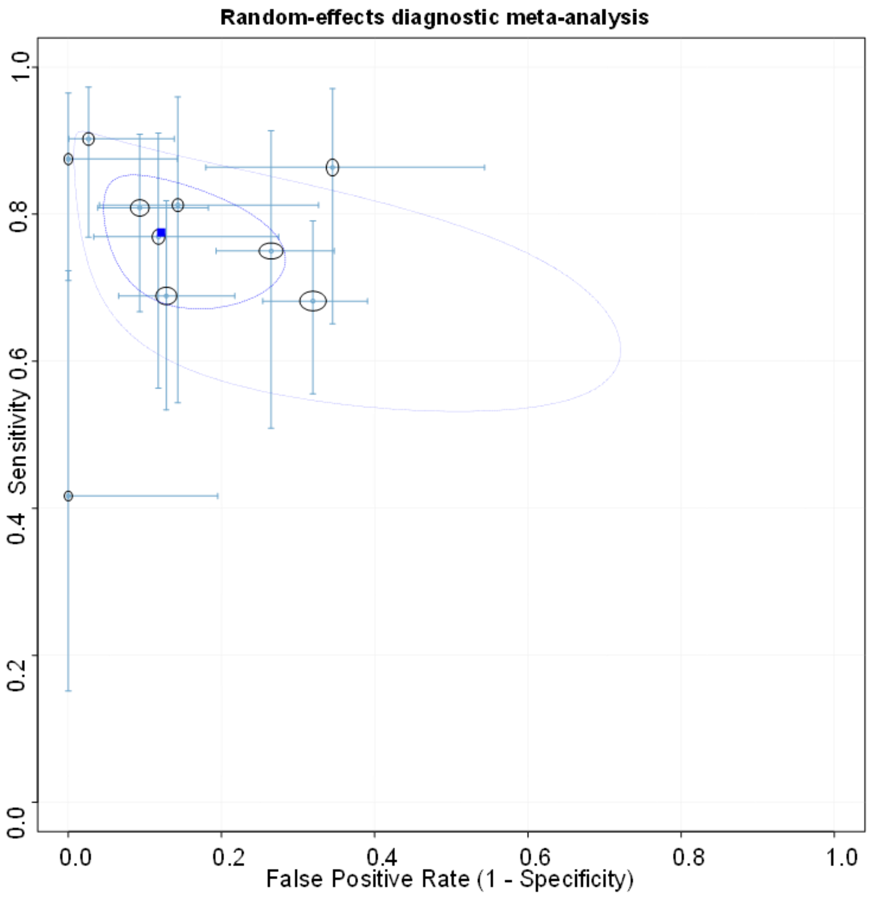

**Figure S3.F. Pooled sensitivity and specificity of MRI**

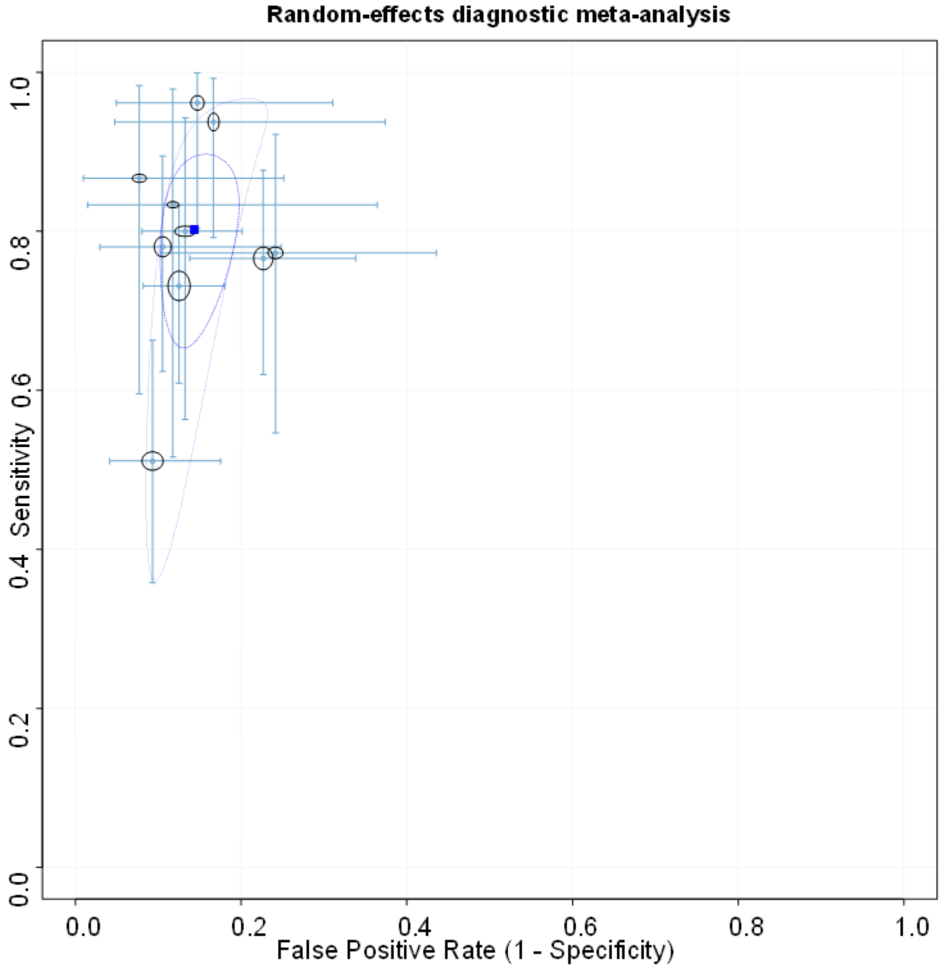

**Figure S4.** Regression plots of sensitivity and specificity over the years

**Figure S4.A.** Sensitivity of TVS over the years

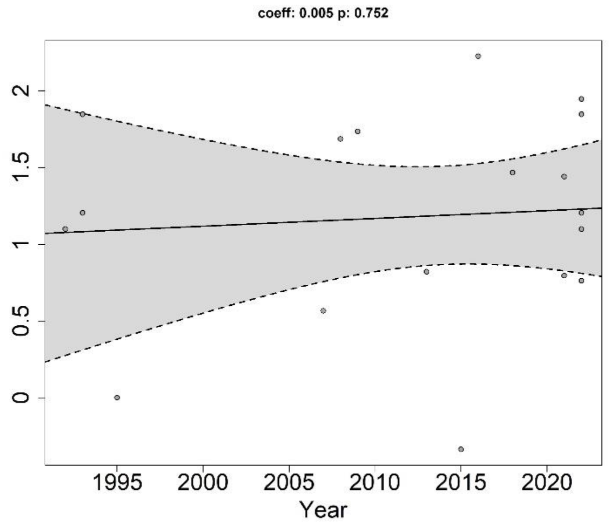


**Figure S4.B.** Specificity of TVS over the years

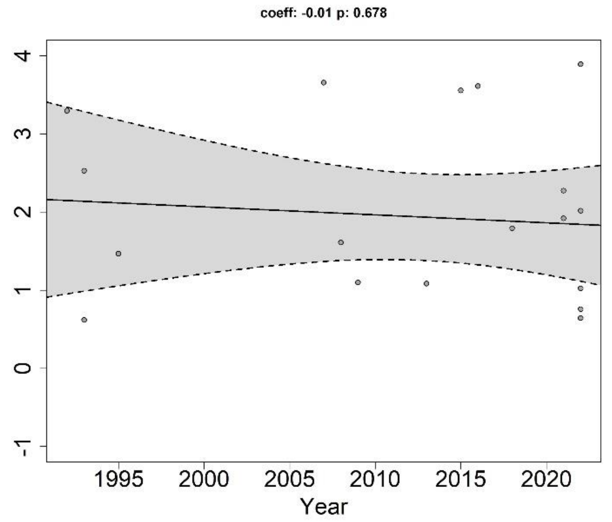


**Figure S4.C.** Sensitivity of MRI over the years

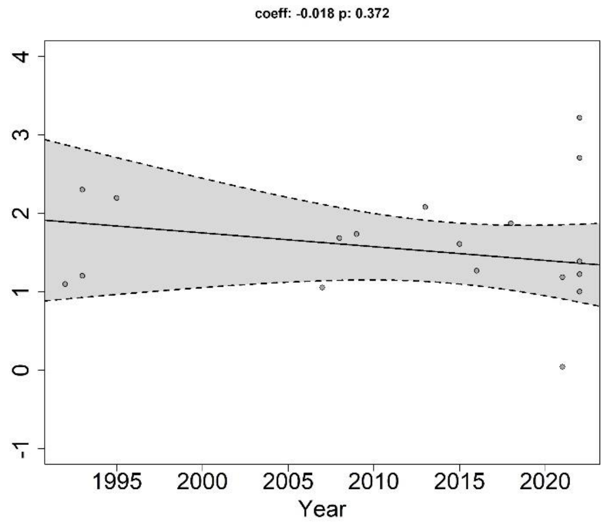


**Figure S4.D.** Specificity of MRI over the years

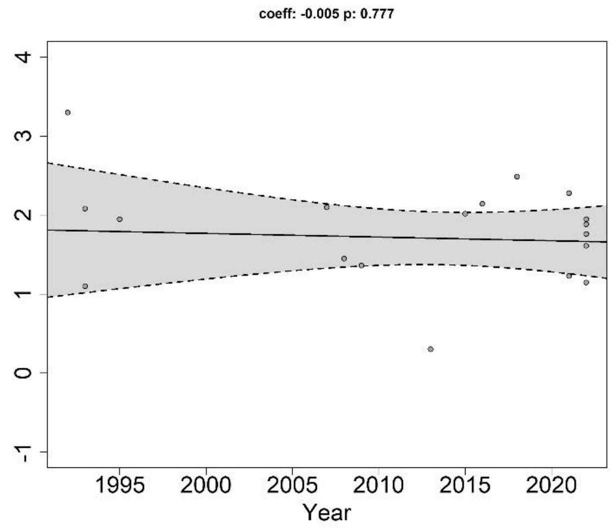

**Figure S5.** Pooled sensitivity and specificity of TVS (A) and MRI (B) in low-grade EC patients

**Figure S5.A. Pooled sensitivity and specificity of TVS**

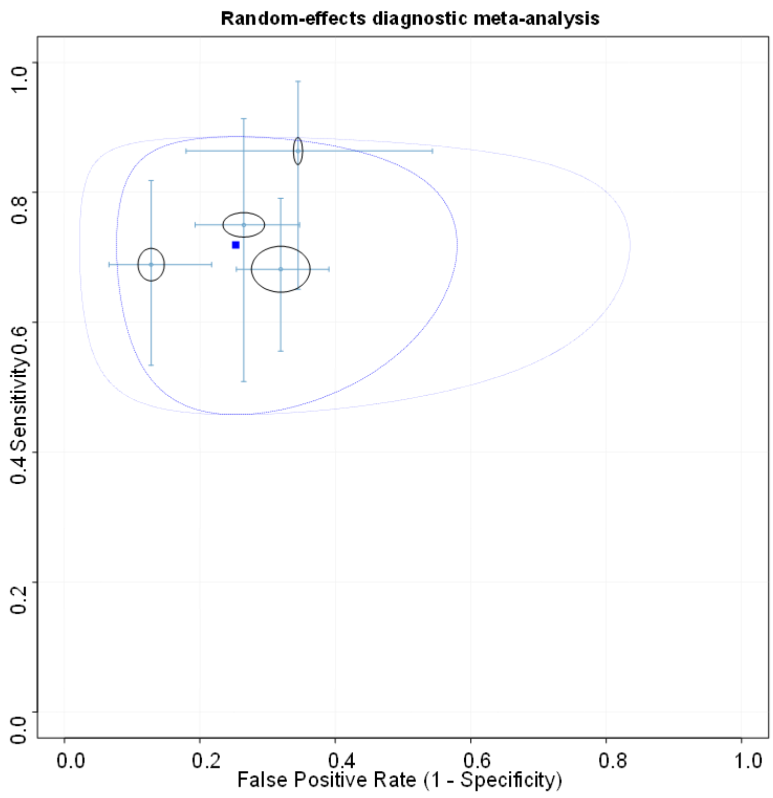


**Figure S5.B. Pooled sensitivity and specificity of MRI**

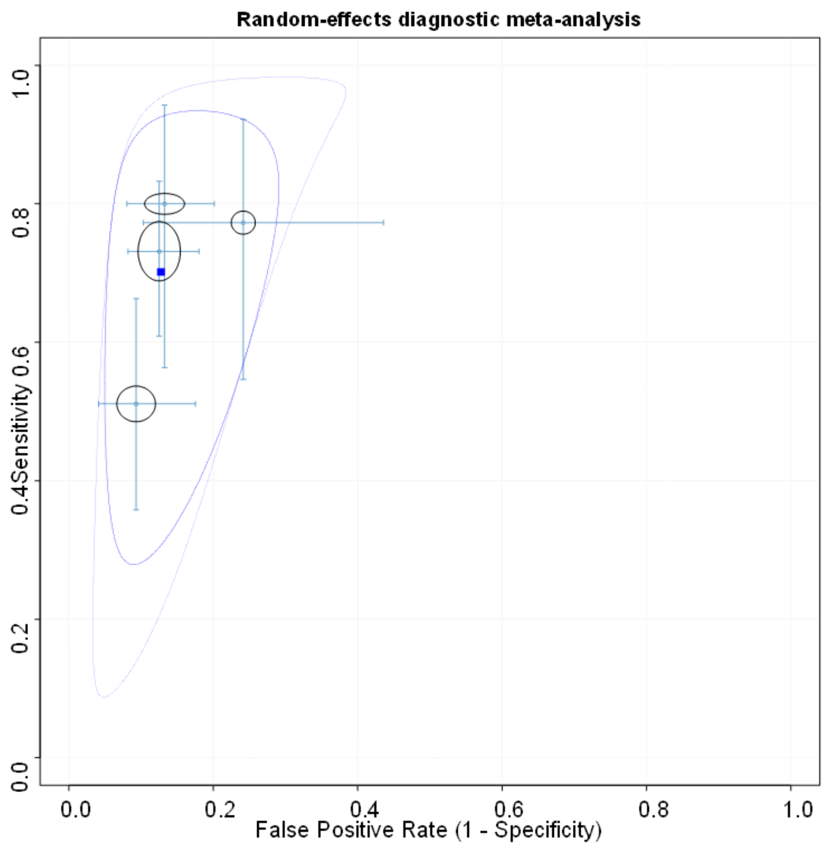

**Figure S6.** MRI data grouped by the sequences used

**Figure S6.A. Forest plot containing the MRI sensitivity data in the T1-T2 group**

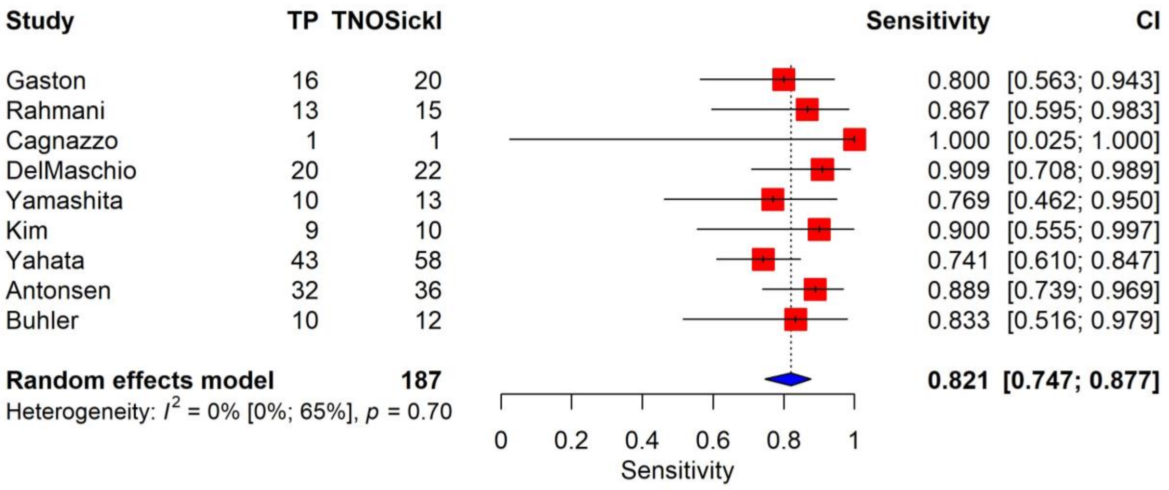


**Figure S6.B. Forest plot containing the MRI specificity data in the T1-T2 group**

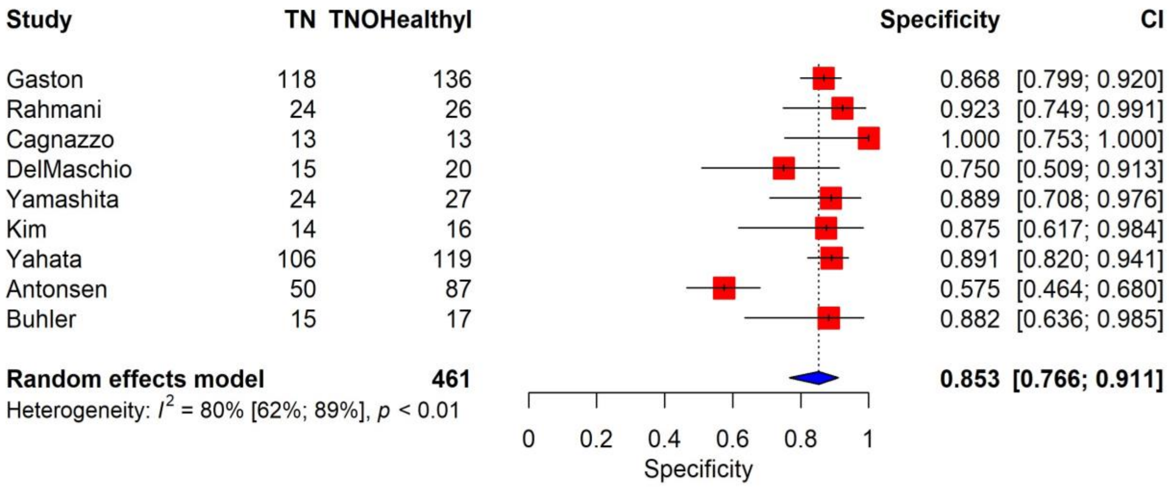


**Figure S6.C. Forest plot containing the MRI sensitivity data in the DCE-DWI group**

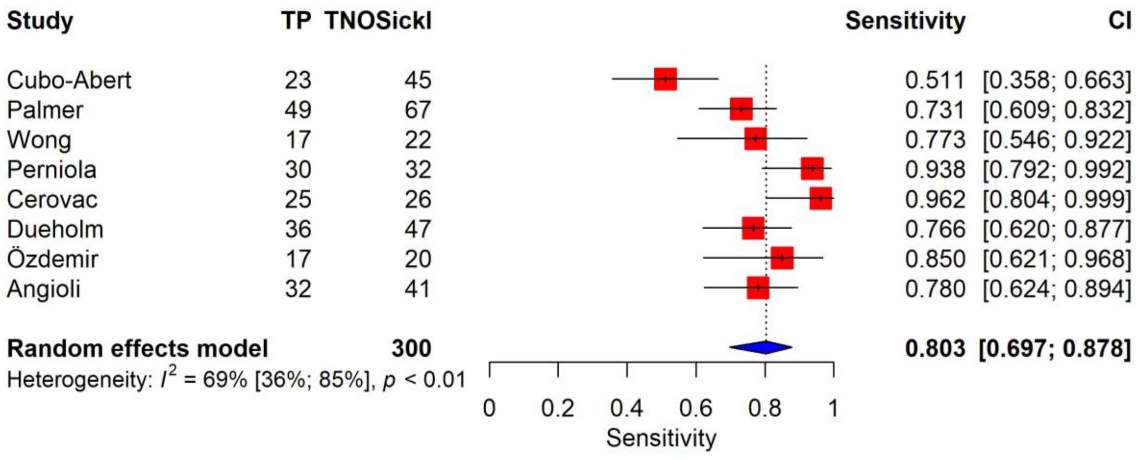


**Figure S6.D. Forest plot containing the MRI specificity data in the DCE-DWI group**

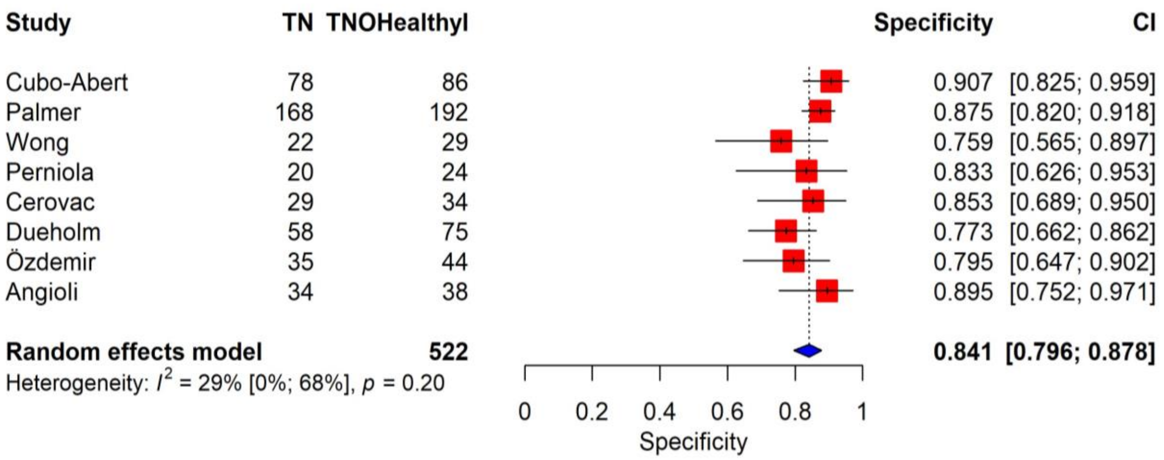


**Figure S6.E. Pooled sensitivity and specificity of the T1-T2 group**

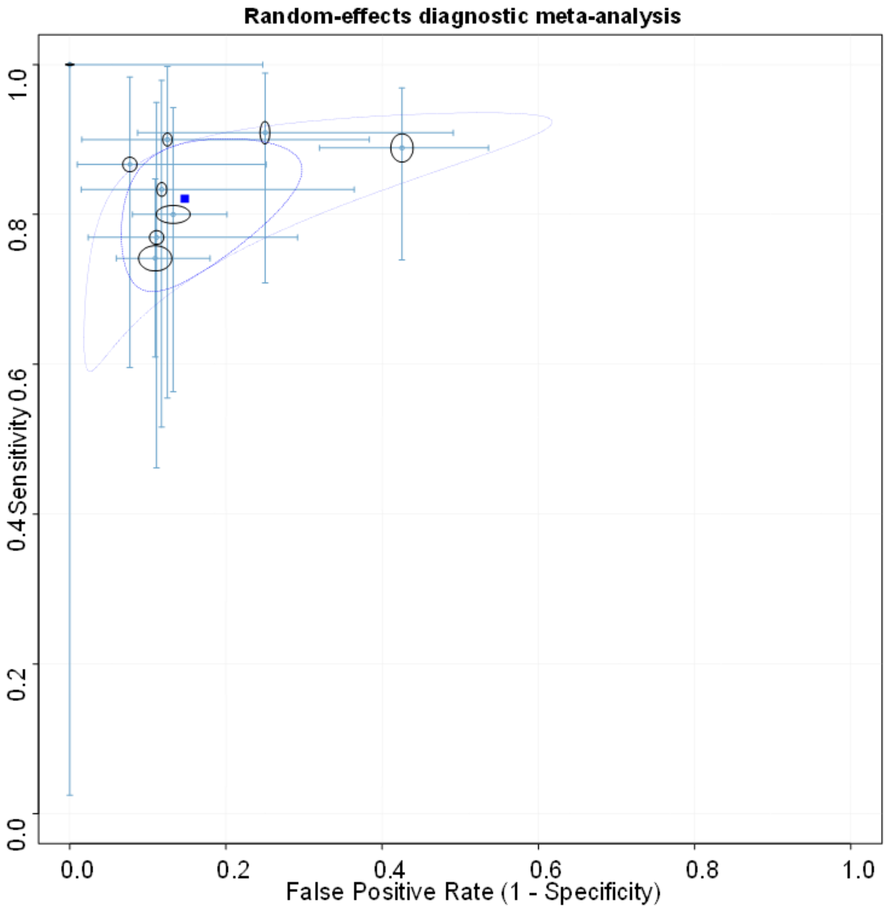


**Figure S6.F. Pooled sensitivity and specificity of the DCE-DWI group**

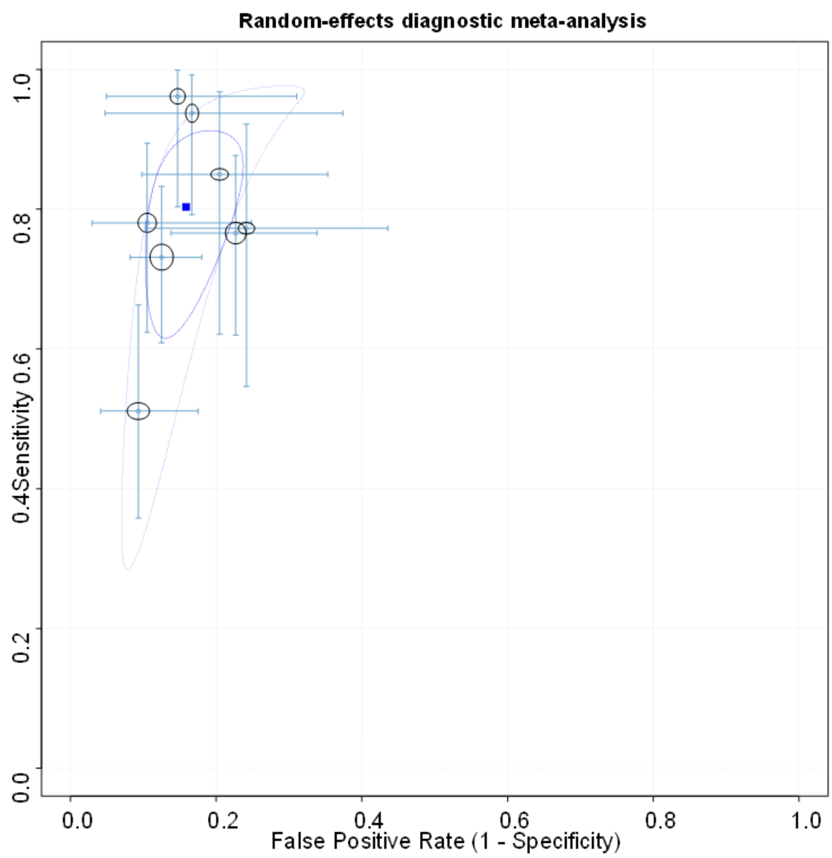


**Figure S7. No myometrial invasion vs. myometrial invasion**

**Figure S7.A. Forest plot containing the data on TVS sensitivity**

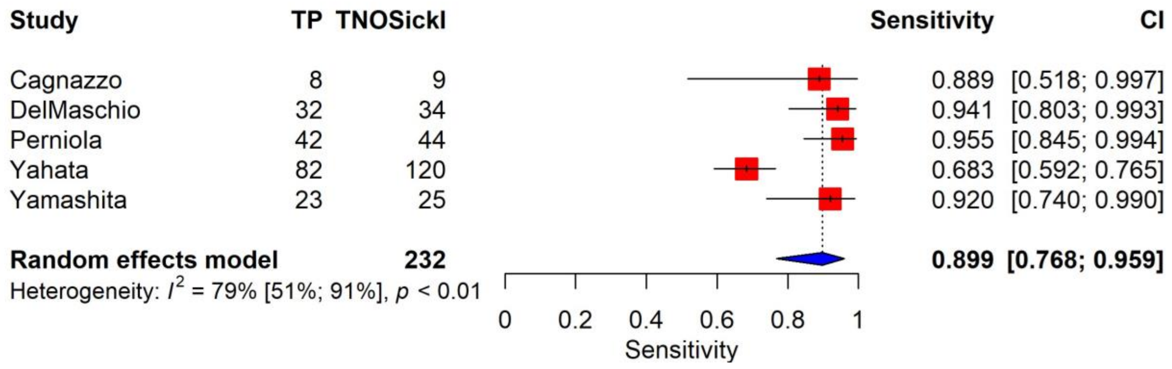


**Figure S7.B. Forest plot containing the data on TVS specificity**

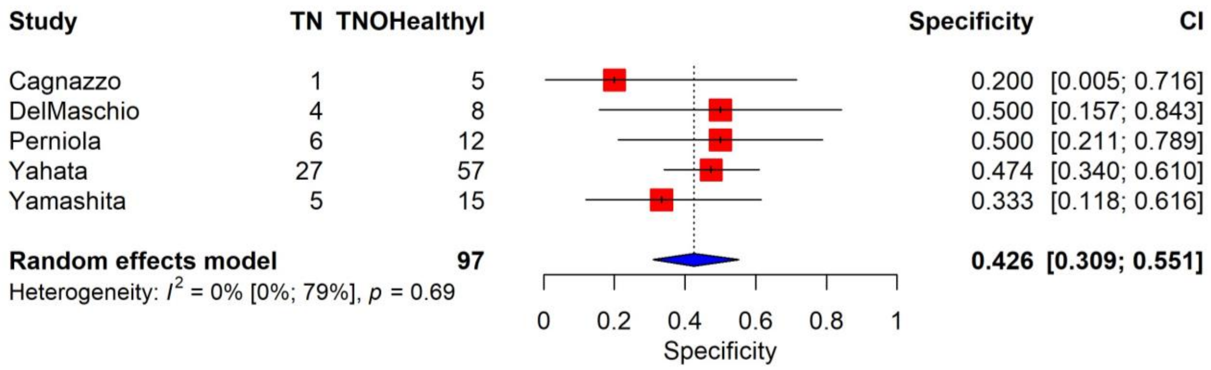


**Figure S7.C. Forest plot containing the data on MRI sensitivity**

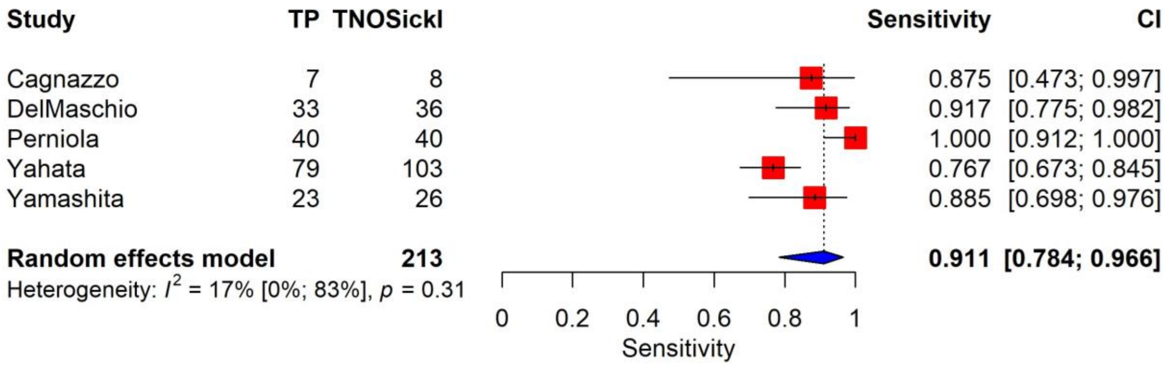


**Figure S7.D. Forest plot containing the data on MRI specificity**

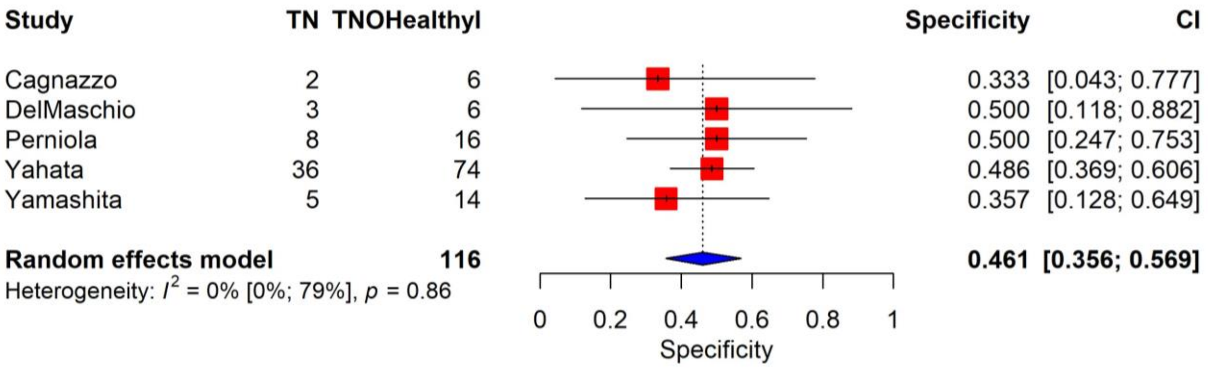


**Figure S8.** Funnel plots, all articles included for TVS (A) and MRI (B)

Figure S8.A. Funnel plot for TVS

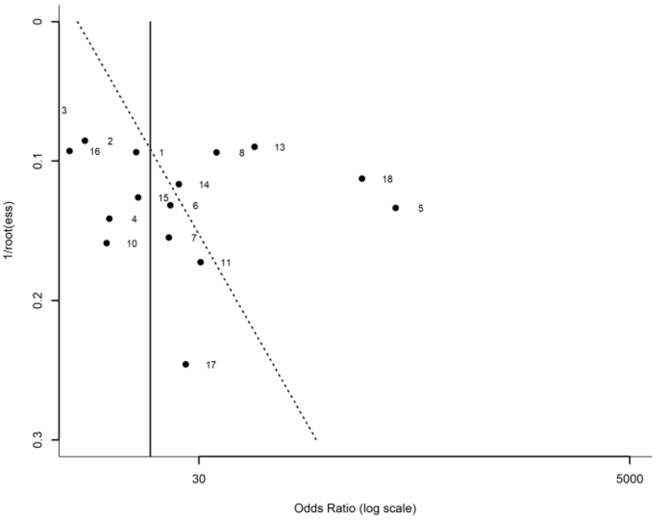

Figure S8.B. Funnel plot for MRI

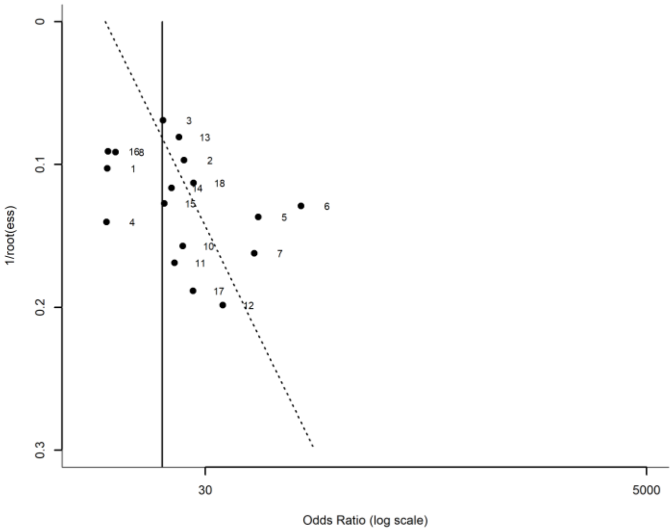

Supplement: Supplementary file 1 [file cancers-16-00907-s001.zip › cancers-2848664-supplementary.pdf]
